# Mutagenesis Screen Identifies *agtpbp1* and *eps15L1* as Essential for T lymphocyte Development in Zebrafish

**DOI:** 10.1371/journal.pone.0131908

**Published:** 2015-07-10

**Authors:** Christoph Seiler, Nichole Gebhart, Yong Zhang, Susan A. Shinton, Yue-sheng Li, Nicola L. Ross, Xingjun Liu, Qin Li, Alison N. Bilbee, Gaurav K. Varshney, Matthew C. LaFave, Shawn M. Burgess, Jorune Balciuniene, Darius Balciunas, Richard R. Hardy, Dietmar J. Kappes, David L. Wiest, Jennifer Rhodes

**Affiliations:** 1 Blood Cell Development and Function Program, Fox Chase Cancer Center, Temple University Health System, Philadelphia, Pennsylvania, United States of America; 2 Translational and Functional Genomics Branch, National Human Genome Research Institute, National Institutes of Health, Bethesda, Maryland, United States of America; 3 Department of Biology, College of Science and Technology, Temple University, Philadelphia, Pennsylvania, United States of America; Rutgers-Robert Wood Johnson Medical School, UNITED STATES

## Abstract

Genetic screens are a powerful tool to discover genes that are important in immune cell development and function. The evolutionarily conserved development of lymphoid cells paired with the genetic tractability of zebrafish make this a powerful model system for this purpose. We used a Tol2-based gene-breaking transposon to induce mutations in the zebrafish (*Danio rerio*, AB strain) genome, which served the dual purpose of fluorescently tagging cells and tissues that express the disrupted gene and provided a means of identifying the disrupted gene. We identified 12 lines in which hematopoietic tissues expressed green fluorescent protein (GFP) during embryonic development, as detected by microscopy. Subsequent analysis of young adult fish, using a novel approach in which single cell suspensions of whole fish were analyzed by flow cytometry, revealed that 8 of these lines also exhibited GFP expression in young adult cells. An additional 15 lines that did not have embryonic GFP^+^ hematopoietic tissue by microscopy, nevertheless exhibited GFP^+^ cells in young adults. RT-PCR analysis of purified GFP^+^ populations for expression of T and B cell-specific markers identified 18 lines in which T and/or B cells were fluorescently tagged at 6 weeks of age. As transposon insertion is expected to cause gene disruption, these lines can be used to assess the requirement for the disrupted genes in immune cell development. Focusing on the lines with embryonic GFP^+^ hematopoietic tissue, we identified three lines in which homozygous mutants exhibited impaired T cell development at 6 days of age. In two of the lines we identified the disrupted genes, *agtpbp1* and *eps15L1*. Morpholino-mediated knockdown of these genes mimicked the T cell defects in the corresponding mutant embryos, demonstrating the previously unrecognized, essential roles of *agtpbp1* and *eps15L1 *in T cell development.

## Introduction

The production of mature immune cells is essential to fight pathogen infections and maintain good health throughout the lifespan of vertebrate animals. Key components of the immune system are T and B lymphocytes. Generation of these cells is dependent upon tightly controlled changes in gene expression that progressively limit the lineage potential of hematopoietic stem cells (HSC) to a single mature hematopoietic lineage [1]. Thus, HSC give rise to the lymphoid lineage, including T lymphocyte precursors that develop in the thymus (T cells) and B lymphocyte progenitors that develop in the bone marrow [1]. While numerous regulators of these developmental processes have been characterized and are highly conserved among zebrafish and mammals [2–4], our knowledge of the networks controlling this process remains limited.

Zebrafish blood cell development is very similar to other vertebrates, with hematopoiesis occurring in several waves [2,4–5]. In zebrafish embryos, the primitive wave originates from the lateral plate mesoderm, and gives rise to *myeloperoxidase* (*mpx*)-expressing myeloid cells and *hbae1-globin*-expressing erythroid cells [6]. The transition to the definitive stage follows the onset of circulation at 25 hours post-fertilization (hpf). Definitive, transient erythromyeloid progenitors arise from the caudal hematopoietic tissue (CHT) while hematopoietic stem cells are derived from the hemogenic endothelium in the ventral wall of the dorsal aorta (VDA) within the aorta-gonad-mesonephros (AGM) region [2,4]. Definitive stem/progenitors in the AGM are marked by *runx1* and *cmyb* expression [2]. Subsequently, *ikaros*-expressing lymphoid progenitors migrate to the thymus and give rise to *rag1-* and *lck*-expressing T cells by 4 days post-fertilization (dpf) [7]. Definitive hematopoiesis moves to the pronephric kidney, the site of adult hematopoiesis. Immunoglobulin (Ig)-μ-expressing B cells are detected starting at 3-weeks of age in the kidney [3]. In contrast to other vertebrate systems, the development of each of these immune cell types can be assessed in live, transparent zebrafish, which emphasizes the utility of this animal model for the study of lymphopoiesis. Accordingly, we performed a genetic screen in zebrafish to identify novel regulators of lymphopoiesis.

We used a gene-disrupting transposon to mutagenize the zebrafish genome and identify genes important in immune cell development [8,9]. Successful integration of the Tol2-based gene trap vector into a gene locus results in direct splicing of upstream coding sequences of the targeted gene to an AUG-less *Gal4-VP16* cassette in the targeting vector, which truncates the transcript. The Gal4 product then activates a reverse oriented UAS element driving eGFP expression [9], thereby fluorescently marking the cells that express the mutagenized gene. This system allowed us to focus our efforts on genes whose expression is enriched in hematopoietic progenitors or mature immune cells. Even low levels of Gal4-VP16 can robustly activate the UAS-driven fluorescent reporter, which allows us to detect genes that are weakly expressed as is typical of regulators of hematopoiesis [9–12]. Historically, chemical-based mutagenesis has been utilized to genetically interrogate the regulatory cascades that control the hematopoietic program in zebrafish, but this requires cumbersome, time-consuming positional cloning to identify the mutated gene [13–24]. The gene trap transposon approach employed here circumvents these pitfalls and allows us to track the fluorescent marker in live embryos, visually identify carriers of the mutation and perform straight-forward identification of the disrupted gene using primers complementary to the integrated trapping vector.

Using a gene trap Tol2 transposon approach, we screened 731 crosses of mutagenized F0 fish and identified 52 gene-trap lines. We assessed GFP marking of hematopoietic cells at embryonic stages and in young adults. Focusing on the embryonic lines, we identified a candidate gene for 8 of the 12 identified gene trap lines, most of which are not known to play a role in blood cell development. Homozygous mutants in 3 of these lines displayed defects in the development of T lymphoid progenitors. The disrupted genes were identified in 2 of the lines as *agtpbp1* and *eps15L1*. Thus, through a genetic screen approach we have identified two previously unrecognized genes that are essential for immune cell development.

## Materials and Methods

### Ethics statement and zebrafish maintenance

All procedures using zebrafish (*Danio rerio*) were performed in accordance with the Guide for the Care and Use of Laboratory Animals by the National Institutes of Health. The protocol was approved by the Institutional Animal Care and Use Committee at Fox Chase Cancer Center, animal welfare assurance statement number A3285, J. Rhodes protocol number 08–7. Zebrafish adults were bred offline and embryos were raised and staged using standard practices [25]. Babies were fed paramecia twice daily; adults were fed twice daily with hatched brine shrimp and once per day with TetraMin flakes. Adults were housed at 1–4 fish per liter of water in an Aquatic Habitats aquarium system, with water parameter set points for conductivity at 621 μS, pH at 7.16 and room temperature at 80°F with a cycle of 14/10 hours of light/dark.

### Mutagenesis using gene trap vector

The syUAS with flanking restriction enzyme sites was synthesized by Integrated DNA Technologies (USA) and then cloned as SpeI-AvrII fragment into *GBT-B1* (pDB783), thus resulting in *GBT-B4* (pDB899). Tol2 cDNA was prepared as described [26] (http://tol2kit.genetics.utah.edu/index.php/Protocols). For the majority of injections, pCS2FA was linearized with NotI, 1ug DNA was transcribed using the SP6 mMessage Machine Kit (Life Technologies) and Tol2 RNA was purified using the Qiagen RNeasy Mini Kit according to manufacturer’s instructions. For a small portion of the injections, pT3TS-Tol2 (pDB600 [27]) was linearized with XbaI and used to make Tol2 mRNA as above. Nomenclature for the gene-trap lines was established by personal communication with the ZFIN database team. The gene-trap construct is named *Gt(LOXP-GAL4-VP16-FRT*, *syUAS*:*EGFP-FRT-LOXP)*, referred to as *GBT-B4*. Here, the lines are referred to by the institute designation “fcc” followed by the line/allele number generated during the screen. To generate F0 founders, 5 nL of a solution containing *GBT-B4* vector DNA (20 ng/μL) and Tol2 mRNA (20 ng/μL) was injected into the cell of 1-cell stage embryos (AB strain). Assuming that integration in somatic cells is a good indication for additional transgene insertions into the germ line, we grew injected larvae that showed strong somatic expression in various tissues, which was about 40% of the total embryos injected.

### Microscopic analysis

Fish were screened on a Nikon SMZ1500 stereomicroscope equipped with X-Cite series 120 fluorescence illuminator and a Digitial Sight DS-Fi1 camera (Nikon). Fluorescence images were taken on the above stereoscope or with a Nikon Eclipse 80i microscope with an Intensilight C-HGF1 fluorescence light source and a DS-Qi1Mc camera using NIS-Elements software (Nikon). Live embryos were mounted in 3% methylcellulose in E3 egg water for imaging. Fixed embryos were mounted in 50%-100% glycerol in PBST for imaging. Brightfield images were obtained on a Nikon SMZ1500 using a SPOT Insight 4 color camera and SPOT Basic software. Images of siblings were obtained one right after the other using identical capture settings and compiled in Adobe Photoshop. Contrast/brightness was adjusted linearly using the dark and bright level slider in Photoshop in a flattened layer containing images of wild-type sibings and mutant larvae. Quantification of fluorescence was obtained from grayscale images that were analyzed using Fiji (ImageJ) [28]. Confocal images were taken on a Nikon Eclipse TE-2000E/C1 Laser Scanning Confocal Microscope using EZ-C1 3.80 software (Nikon).

### Flow cytometric analysis

Clutches of 6 week old euthanized juvenile fish were manually dissociated between frosted glass microscope slides, passed through 70 micron Nitex cloth filter, resuspended in staining medium (Deficient RPMI, 3% newborn calf serum, 0.1% sodium azide). Cells were pelleted by centrifugation at 1200 rpm (273 x G), for 7 minutes. The pellet was resuspended in 1 ml staining medium and layered over 1 ml Lympholyte M (Accurate Scientific) and centrifuged 20 minutes at 2800 rpm (1762 x G). Cells were recovered from the interface and washed two times with staining medium. Cell pellets were then resuspended in staining medium, propidium iodide (1ug/ml) was added and the cells were transferred to Falcon 2054 tubes. Samples were sorted on a Becton-Dickinson FACS Vantage SE or an Aria II cell sorter. GFP^+^ cells were processed as previously described [29].

### Identification of transposon insertion site or disrupted gene

For DNA preps, larvae or fin clips were digested (10mM Tris, 2mM EDTA, 0.2% Triton X, 0.2mg/ml Proteinase K; pH 8.0) at 55°C overnight. The proteinase K was inactivated at 98°C for 15 minutes prior to PCR. For RNA preps, pools of 1–10 larvae were mechanically disaggregated with a 20-gauge needle and RNA was purified using a Nucleospin RNA kit (Macherey-Nagel). RACE was performed using SMARTer RACE Kit (Clontech) following the manufacturers instructions. Primers are listed in S1 Table. RACE products were cloned into TOPO vector and sequenced. iPCR was performed as described [9], with minor modifications. Genomic DNA was isolated from pools of 10 embryos at 5 dpf using the Qiagen genomic DNA isolation kit and digested with both BamH1 and Bgl-II (NEB). Ligation was performed overnight at 16°C with T4 DNA ligase (NEB). PCR was performed using Platinum Hifi polymerase (Life Technologies). Sequences were analyzed using ApE plasmid editor (http://biologylabs.utah.edu/jorgensen/wayned/ape/) and Ensemble (http://www.ensembl.org/Multi/blastview). Linkage analysis was performed for insertion sites identified by iPCR or parallel sequencing (lmPCR). Integration sites were mapped by high-throughput sequencing as previously described [30], with the following modifications: The first round of PCR was performed using Tol2 ITR primer 5’-AATTTTCCCTAAGTACTTGTACTTTCACTTGAGTAA-3', linker primer 5’- GTAATACGACTCACTATAGGGCACGCGTG-3' using the following cycle conditions: 95°C 2 minutes, 25 cycles of: 95°C 15 seconds, 55°C 30 seconds, 72°C 1 minute. PCR amplicons were diluted 1:50 and a second round of PCR was performed using a nested Tol2 ITR primer- 5’-TCACTTGAGTAAAATTTTTGAGTACTTTTTACACCTC-3', nested linker primer- 5’-GCGTGGTCGACTGCGCAT-3' with following cycle conditions: 95°C 2 minutes, 20 cycles of: 95°C 15 seconds, 58°C 30 seconds, 72°C 1 minute. PCR amplicons from the second round were pooled and the sequencing library was prepared for Illumina Miseq sequencing platform. Integrations were recovered from parallel sequencing using a version of GeIST software modified to work with Tol2 vectors [30]. For linkage analysis, F1 or F2 fish were crossed to AB wildtype fish and genomic DNA from 6 GFP positive and 6 negative larvae were tested by PCR for inheritance of the insertion. Insertions identified by RACE were tested by RT-PCR for expression of (*target–gal4*) fusion transcripts using 6 positive larvae and 2 pools of 3 negative embryos. Primers are listed in S1 Table. Full-length gels are shown in S1 and S2 Figs.

### Morpholinos and RT-PCR

Morpholinos diluted in water were injected as previously described [31]. The morpholino sequences are listed in S1 Table. The *agtpbp1* morpholino targets the 3’ splice junction of exon 4 and the *eps15L1* morpholino targets the 3’ splice junction of exon 13. The *p53* morpholino (MO4) sequence is published [32]. The knockdown efficacy of the morpholinos was tested by obtaining RNA from pools of embryos, as described above, and using the Onestep RT-PCR kit (Qiagen). Equal amounts of RNA were used per experimental condition and the corresponding control sample. The RT-PCR primers to test expression of *eps15L1* and *agtpbp* are listed in S1 Table. For semiquantitative RT-PCR, RNA was obtained from purified cells or whole embryos and the RT-PCR was performed as described above. The primers used to detect expression are listed in S1 Table. Greyscale images of the amplified bands were analyzed using Fiji and normalized against *ß*-*actin*. The original gels images are shown in S1 and S3 Figs.

### Whole mount in situ hybridization

Whole mount in situ hybridization was performed as described [31,33] at 58°C. Generation and use of the *lck* and *rag1* probes has been previously described [33]. Riboprobes for *ubap1*, *agtpbp1* and *eps15L1* in pCRII were linearized by digestion and transcribed to RNA probes as follows: *eps15L1*: BstX1 or Kpn1/T7 RNA polymerase for antisense probe and Not1 or EcoRV/SP6 for sense; *agtpbp1*: BamHI/T7 for antisense and NotI/SP6 for sense; *ubap1*: BamHI/T7 for antisense and NotI/SP6 for sense. RNA probes were generated with digoxigenin RNA labeling mix (Roche) and purified by NucAway Spin Columns (Ambion). Staining was developed with Vector labs BCIP/NBT substrate kit according to manufacturer’s instructions. Transverse sections were obtained by manually slicing fixed, stained embryos used for WISH analysis.

## Results

### Tol2-based mutagenesis

**Fig 1:**
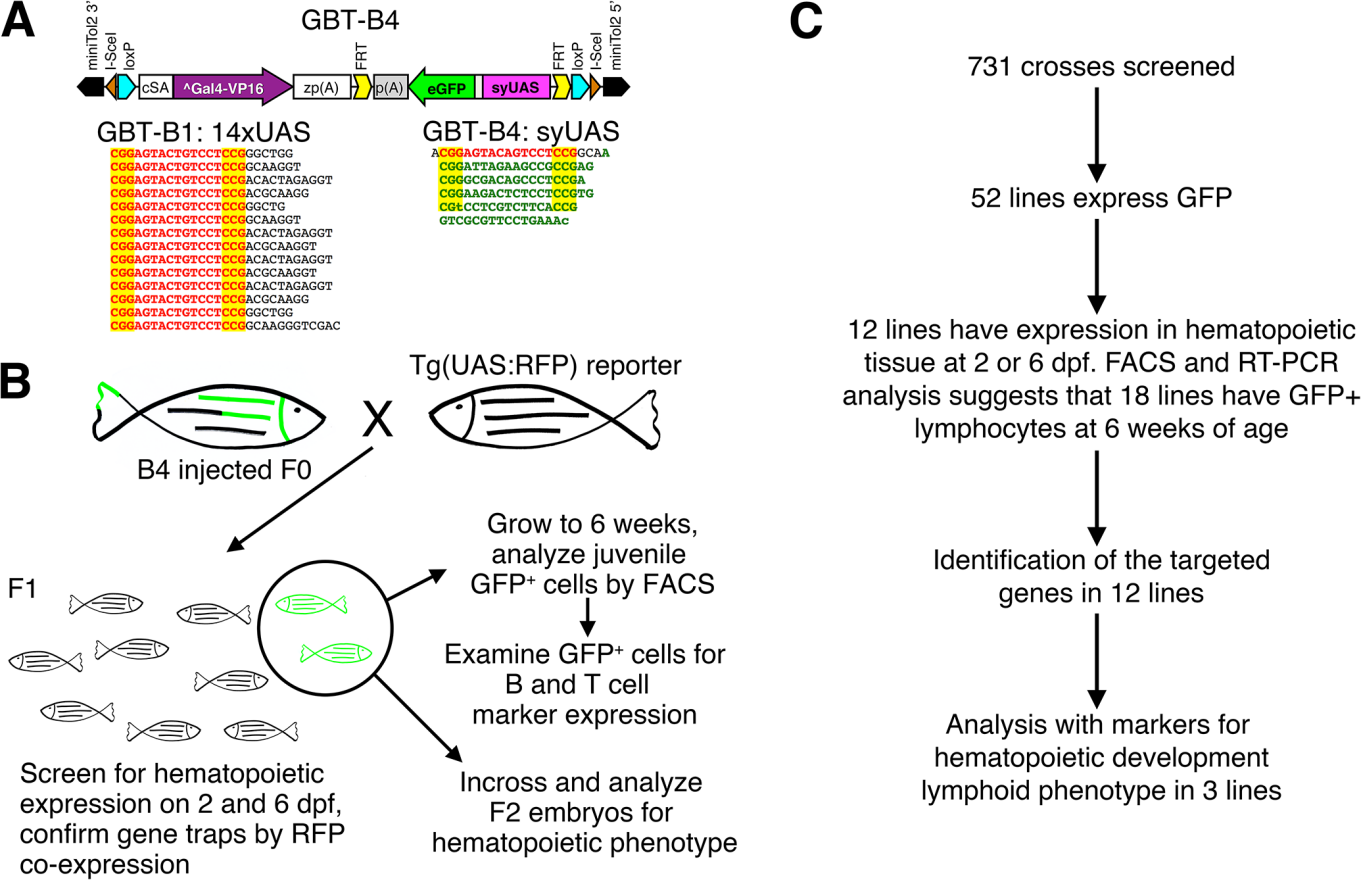
The *GBT-B4* genetic screen approach and results. (A) Design features of the *GBT-B4* gene trap and the nucleotide sequence of the 14XUAS used in the parental *GBT-B1* and the 4.5xUAS syUAS engineered of *GBT-B4*. The consensus Gal4 binding site based on the 14XUAS is in red bold letters, sequences based on the Saccharomyces UAS are in bold green. The core Gal4 binding site CGGN_11_CCG is highlighted in yellow. The non-consensus nucleotide in the 4^th^ binding site of the syUAS is in lower case (t instead of G). (B) Diagram of the genetic screen; arrows indicate the workflow. (C) Summary of the screen results.

To identify new genes that are expressed in hematopoietic cells and are important for development of the immune system, we used a Tol2 transposon-mediated gene trap to create a panel of mutant lines with random insertions in the zebrafish genome. The *G*ene *B*reaking *T*ransposon—*B*ipartite 4 (GBT-B4) contains a potent splice acceptor upstream to an AUG-less Gal4-VP16 and a reverse oriented *UAS*:eGFP cassette (Fig 1A), which effectively prevents expression of downstream exons of the affected gene [9,34,35]. The GBT-B4 vector is very similar to the recently published GBT-B1 vector [9,36]. To alleviate silencing of the 14X*UAS* cassette [37,38], we replaced the highly repetitive synthetic 14X*UAS* with a *UAS* based on the *GAL1-10* locus of the yeast *Saccharomyces cerevisiae* [39,40]. The yeast element contains three complete Gal4 binding sites and a fourth site with a single nucleotide change (CGTN_11_CCT instead of CGGN_11_CCT). This divergent binding site is only bound by Gal4 cooperatively when the other three sites are present [40]. Since a minimum of 4–5 Gal4 binding sites are needed for full Gal4-*UAS* activity in zebrafish [10,41,42], we added an additional Gal4 binding site to the yeast-based sequence, resulting in a hybrid synthetic/yeast-based *UAS* (*syUAS*) with 5 Gal4 binding sites (Fig 1A). Lines generated with this modified construct may be less likely to undergo transposon silencing in successive generations.

Successful insertion of the gene trap vector within a transcribed gene results in the fusion of the flanking 5’ exon of the targeted genes to the Gal4 cassette, and truncation of the transcript. *Gal4* drives expression from the *UAS*:eGFP cassette, which functions to report the expression pattern of the disrupted gene by marking those cells with GFP, which is readily detectable both by immuofluorescence microscopy and flow cytometry. In some cases, however, the GFP reporter may be expressed by direct activation of its minimal promoter by an adjacent enhancer (termed enhancer trap, exampled in [43]). When this happens the *Gal4* gene will not be expressed, therefore, GFP^+^ enhancer trap lines can be distinguished from true GFP^+^ gene trap lines by testing for expression of Gal4 through crosses to a *Tg(UAS*:*RFP)* reporter line [9,36].

### Identification of hematopoietic gene-trap lines

To generate a panel of F0 mutant founders for our screen, we co-injected GBT-B4 with Tol2 transposase mRNA into 1-cell zebrafish embryos. Larvae that exhibited strong GFP expression in any tissue at 6 days post fertilization (dpf), indicative of successful transposon incorporation into the genome, were grown to adulthood. To identify founders with germ line transmission, F0 male adults were outcrossed to Tg(*UAS*:mRFP) reporter females (Fig 1B). Embryos were visually screened for both GFP and RFP expression by fluorescence microscopy at 2 and 6 dpf. Due to the mosaic nature of germline integration in F0 larvae, only 5–20% of the F1 offspring exhibited GFP expression. The GFP^+^ F1 larvae were grown to adulthood to establish stable lines, which thereafter inherited the insertion in roughly the expected Mendelian frequencies. In total we screened offspring from 731 injected F0 fish and identified 52 GFP^+^ lines (Fig 1C and S2 Table).

**Fig 2:**
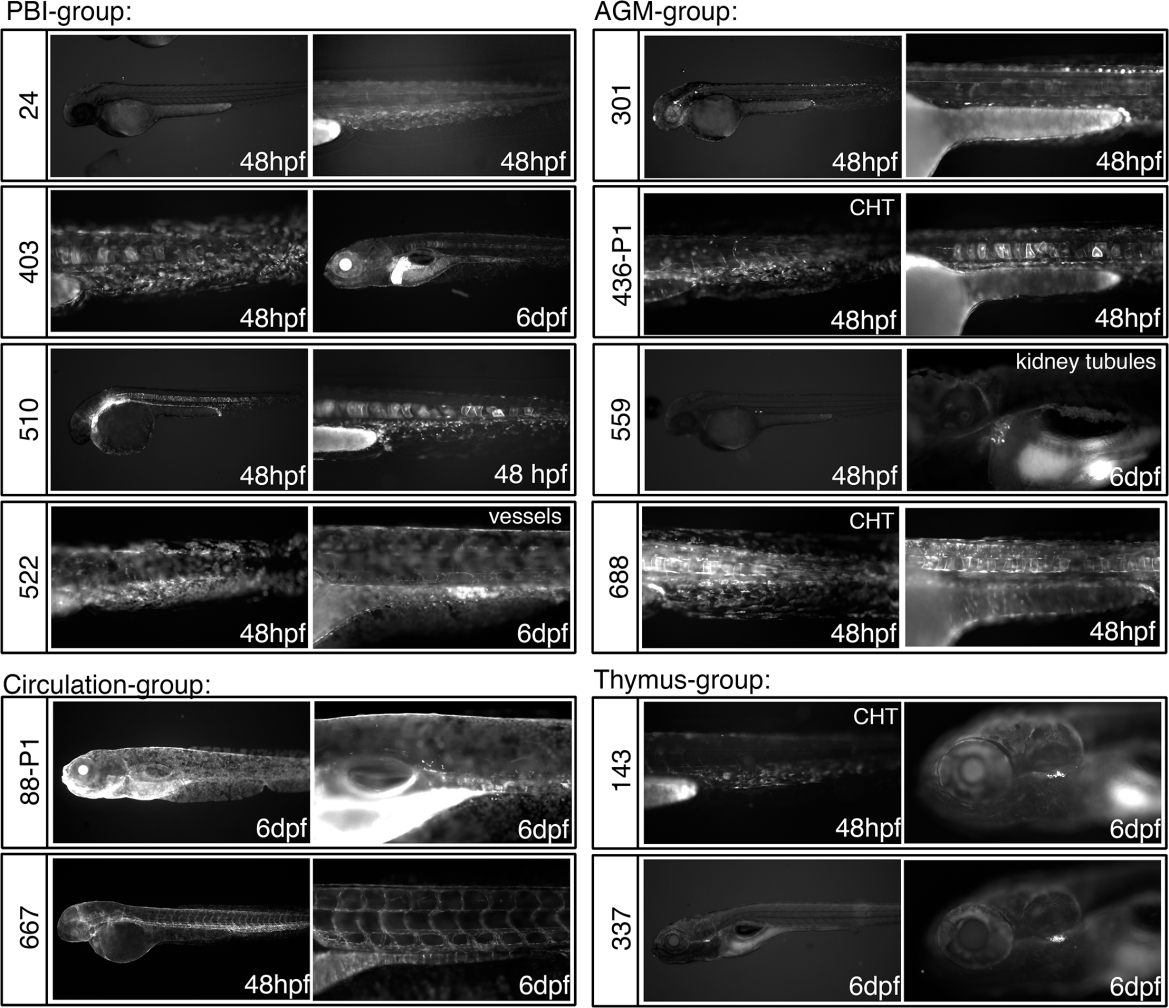
Patterns of GFP expression in *GBT-B4* gene trap lines that include hematopoietic tissues. Lateral views of embryos at 48 hpf or 6 dpf are shown. The *Tg(GBT-B4)fcc* line number is indicated to the left of the panels. The embryo age is indicated. CHT = caudal hematopoietic tissue; AGM = aorta-gonad-mesonephros. Note that the lines are grouped by hematopoietic expression patterns, but the embryos can express GFP in additional tissues.

To identify lines that express the gene-trap reporter in hematopoietic tissues, GFP fluorescence was examined in F1 larvae at 2 and 6 dpf by fluorescence microscopy. At 2 dpf, definitive hematopoietic progenitors are present in the aorta gonad mesonephros (AGM) region and the posterior blood island (PBI), while primitive erythrocytes are found in circulation [44]. Differentiated definitive hematopoietic cells are detectable in the thymus and in circulation by 6 dpf [44]. We identified 12 lines with GFP expression in hematopoietic tissue at 2 or 6 dpf (Fig 2): 4 gene trap lines expressed GFP in the PBI, 4 lines showed expression in the AGM, 2 lines displayed GFP^+^ circulating cells and 1 line expressed GFP in cells that surrounded *Tg(rag2*:*mCherry)* lymphoid cells in the thymus, suggesting that the GFP marks the thymus epithelium (S4A Fig). An additional line displayed GFP^+^ cells in the thymus that co-expressed the *rag2*:*mCherry* transgene (S4B Fig) but was determined to be an enhancer trap (Fig 2, line *fcc337*). Most of the hematopoietic gene-trap lines show expression in additional hematopoietic and/or non-hematopoietic tissues (Fig 2, see Table 1 for complete description).

**Table 1:** Summary of lines with embryonic marking of hematopoietic tissue and identification of the disrupted genes.

Line*	Expression	Gene	Entrez ID	Method	Chr.	Position	Strand	Exon rank	% cDNA inhibition
*Tg(GBT-B4)fcc24*	2dpf: PBI; 2,6dpf: circulation	*ralgds*	799483	lmPCR	8	31822987	-	2	n.d.
*vps4b* ^*fcc88-P1Gt*^ *; Tg(GBT-B4)fcc88-P1*	2/6dpf: skin, kidney, circulation	*vps4b*	393880	lmPCR	2	13060212	-	2	0.68
*Tg(GBT-B4)fcc143*	2dpf: AGM, PBI; 6dpf: thymus epithelium	*(ubap1)*	28279726	RACE	21	n.d.	n.a.	6	0.56
*agtpbp1* ^*fcc301Gt*^ *; Tg(GBT-B4)fcc301*	2/6dpf: kidney, nerves, circulation	*agtpbp1*	65335310	RACE	8	n.d.	n.a.	2	n.d.
*Tg(GBT-B4)fcc337-P2*	6dpf: thymus	n.a.				n.d.			enhancer trap
*vps35* ^*fcc403Gt*^ *; Tg(GBT-B4)fcc403*	2dpf: PBI, notochord; 6dpf: blood vessels, circulation	*vps35*	561697	lmPCR	7	44541300	-	2	0.87
*eps15L1* ^*fcc436-P1Gt*^ *; Tg(GBT-B4)fcc436-P1*	2dpf: vessels, PBI, AGM, hatching gland skin; 6dpf: pancreas, intestine, kidney, skin	*eps15L1*	528472310	RACE	2	n.d.	n.a.	2	0.71
*Tg(GBT-B4)fcc510*	2dpf: PBI, notochord kidney; 6dpf: notochord, kidney, enteric nervous system	n.d.							
*Tg(GBT-B4)fcc522*	2dpf: skin, PBI; 6dpf: vessels, intestine, circulation	n.d.							
*adamts3* ^*fcc559Gt*^ *; Tg(GBT-B4)fcc559*	2dpf: cells in AGM; 6dpf: cells in anterior kidney	*adamts3*	568788	lmPCR	5	47079485	+	15	0.94
*abi1a* ^*fcc667Gt*^ *; Tg(GBT-B4)fcc667*	2dpf: blood vessels, PBI; 6dpf: vessels, circulation	*abi1a*	393711	lmPCR	24	6019688	+	2	0.71
*Tg(GBT-B4)fcc688*	2dpf: PBI, skin, kidney nose, notochord, 6dpf: skin, kidney, spinal chord, hair cells (ear)	*hnrpkl*	406264	lmPCR	5	56989741	-	11	n.d.

*GBT-B4 = *Gt(LOXP-GAL4-VP16-FRT*, *syUAS*:*EGFP-FRT-LOXP)*

### Identification of GFP marked lymphoid cells in juvenile fish

We wanted to assess the gene-trap mediated marking of cells in fish older than 6 dpf, at an age when mature T and B cell lymphocytes are present. B lymphocytes are detected in the kidney with probes for Immunoglobulin (Ig)-μ and Ig light chain and T lymphocytes in the thymus with a probe for T-cell antigen receptor alpha chain constant region (TCRAC) at 3 weeks of age [3,45,46]. The dorso-ventral extension of the thymus occurs in juvenile fish through 6 weeks of age [46], and roughly coincides with the ability of fish to produce an antibody response following immunization with human gamma globulin or formalin-killed bacteria [45]. Thus, we decided to assess the GFP^+^ cells in 6-week old fish. However, the detection of GFP^+^ cells by microscopy is challenging in juvenile fish compared to embryos, due to decreased transparency and increased body mass. Moreover, immune tissues include stromal and hematopoietic components and the analysis of fish by microscopy does not always allow one to cleanly distinguish GFP expression in cells of hematopoietic origin from the supporting stroma. Therefore, we used fluorescence activated cell sorting (FACS) and reverse transcription polymerase chain reaction (RT-PCR) analysis to both determine if the gene-trap marked cells were of hematopoietic origin, since such cells should be liberated from embryos by mechanical disruption, and to determine the lineage of those cells, as determined by expression of lineage markers.

**Fig 3:**
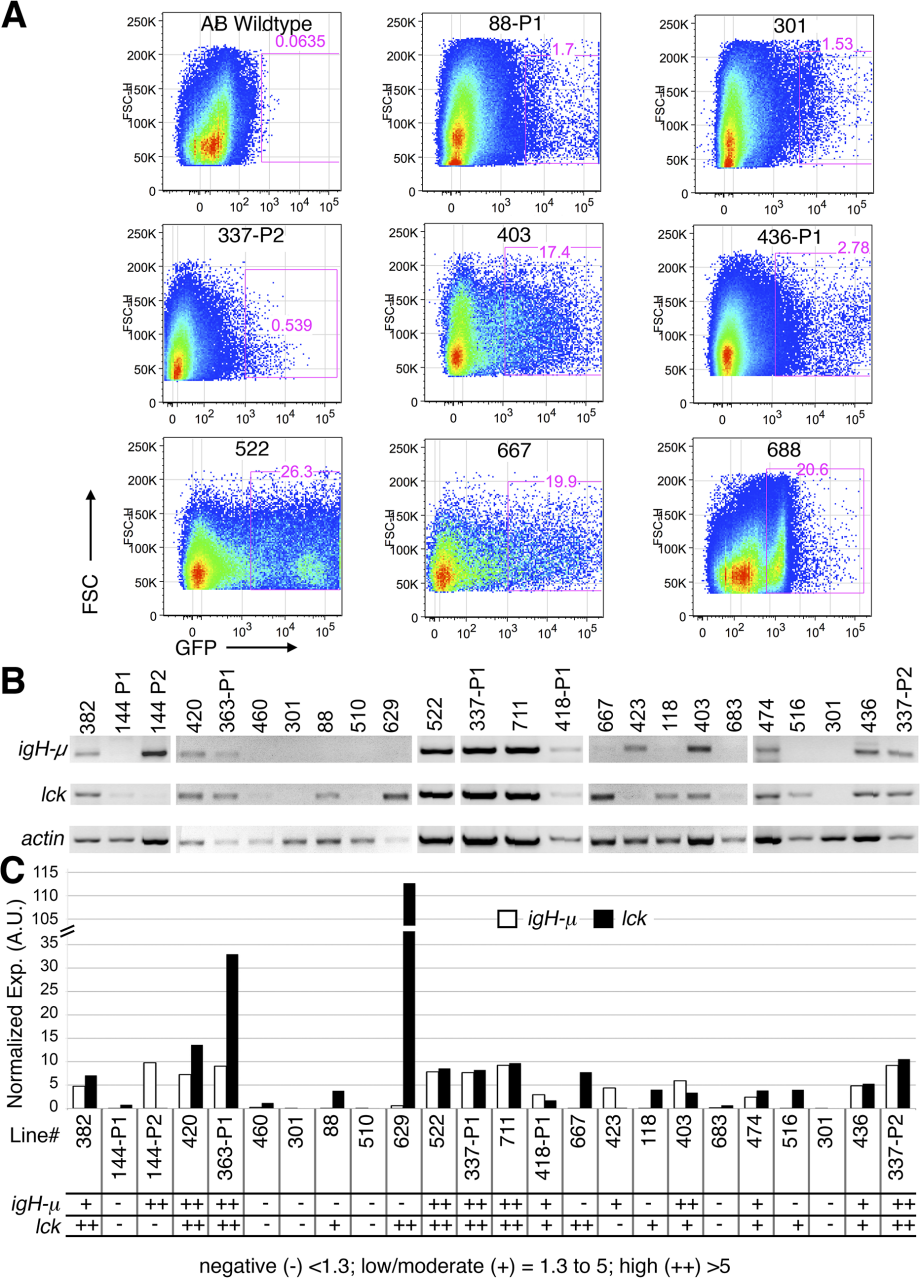
Identification of GFP populations that express T and B cell markers in young adult fish. (A) Examples of scatter plots from fluorescence-activated cell sorting (FACS) of GFP+ cells from the indicated *Tg(GBT-B4)fcc* lines. Plots show GFP versus forward scatter (FSC, indicates cell size). The AB wildtype is a negative control. The line name and percent positive GFP cells out of the total events is indicated for each plot. (B) RT-PCR analysis of *igH-*μ, *lck* and *ß-actin* in GFP^+^ cells from the indicated Tg(GBT-B4)fcc lines. Lanes lacking an *actin* signal are not shown; vertical separation indicates independent experiments. Duplicates of fcc301 are shown (B-C). (C) Quantification of *igH-*μ and *lck* expression in GFP^+^ cells from the indicated lines. A.U. = arbitrary units. The scoring scale is listed below the results.

To identify lines that express GFP in hematopoietic cells at 6 weeks of age, we crossed F0 fish as above, grew at least 30 F1 larvae and analyzed single cell suspensions from manually disaggregated clutches by FACS. The gating strategy selected cell populations based on forward and side light scatter parameters determined to enrich for lymphocytes, as well as GFP expression. The GFP^+^ populations were defined by comparison to age matched, non-transgenic control fish (Fig 3A). In preliminary analysis of the first 200 crosses, we determined that flow cytometric detection of GFP^+^ cells released from 6 week old juvenile fish was only observed in lines that also exhibited GFP expression at 2 or 6 days by microscopy. Therefore, we focused subsequent FACS analyses on gene trap lines that exhibited GFP expression in any tissue at 2 or 6 days. We detected GFP^+^ cells in 23 of the 52 lines analyzed at 6 weeks (Fig 3 and S2 Table). Of note, 15 lines that contained GFP^+^ cells in juvenile fish did not display embryonic hematopoietic GFP expression in 2 and 6 dpf embryos, suggesting that these lines have inserts in genes expressed only in adult blood cells or that the expression of GFP was too low to be detected by microscopy. Thus, FACS, which integrates the fluorescent signal from an entire cell, may provide greater sensitivity than microscopy. The embryonic expression and flow cytometric analysis for the FACS-GFP^+^ lines is summarized in S2 Table.

Since the GFP^+^ population from the gene trap lines could be comprised of a variety of cell types, we examined the expression of B and T cell genes in purified GFP^+^ cells to determine whether the cell population marked by the gene-trap vector included lymphocytes. This analysis was performed on all lines in which sufficient GFP^+^ cells could be purified by flow cytometry to permit detection of a *ß-actin* control signal. RT-PCR analysis of *IgH-*μ (for B-cells) and *lck* (T-cells) within sorted GFP^+^ populations showed expression of lymphoid gene transcripts in 18 of the 23 total lines. In sum, 5 lines contained T- but not B- cells; 2 lines contained B- but not T-cells; 11 lines showed expression of both the B and T cell markers and 5 lines were negative for both markers (Fig 3B and 3C). This suggests that the transposon insertion in these lines likely disrupts genes expressed in these subsets of cells, although our analysis does not rule out expression in other cell types.

### Target gene identification in gene trap lines

We used 3 approaches to identify the disrupted gene in the gene trap lines: (1) inverse PCR (iPCR) [9,47], (2) linker-mediated PCR (lmPCR) followed by high-throughput sequencing [29,48] and (3) rapid amplification of cDNA ends (RACE). We favored using inverse and linker-mediated PCR approaches because this enables us to identify not only the disrupted gene, but also the integration site of the gene-trap vector, thereby enabling genotyping of mutagenized fish. We identified a disrupted gene predicted to produce an in-frame fusion with Gal4 in 8 of the 11 gene trap lines that had expression in hematopoietic tissues at day 2 or 6. Next, we determined whether GFP expression was linked to the identified genomic insertion by assessing gene disruption in 6 individual GFP positive embryos and 6 GFP negative siblings of each line (S1 and S2 Figs). These results are summarized in Table 1. The disrupted gene in each line are as follows: *fcc24*: *ral guanine nucleotide dissociation stimulator (ralgds)*, *fcc88-P1*: *vacuolar protein sorting 4 homolog B (vps4b)*, *fcc301*: *ATP/GTP binding protein 1 (agtpbp1)*, *fcc403*: *vacuolar protein sorting 35 homolog (vps35)*, *fcc436-P1*: *epidermal growth factor receptor pathway substrate 15-like 1 (eps15L1)*, *fcc559*: *ADAM metallopeptidase with thrombospondin type 1 motif*, *3 (adamts3)*, *fcc667*: *abl-interactor 1a (abi1a)* and *fcc688*: *heterogeneous nuclear ribonucleoprotein K (hnrpkl)*. We found that GFP expression in line *fcc143* is linked to an insertion in the gene *ubap1*, but we could not confirm that the *ubap1* gene-trap causes the GFP expression (see below).

Since the gene breaking transposon decreases expression of full-length endogenous transcripts, we used semiquantitative RT-PCR to evaluate the level of endogenous target gene expression. For most of the identified lines we could separate 25% of the siblings by high GFP expression, which likely reflects homozygosity for the gene trap insertion. RT-PCR analysis using exon-spanning primers downstream of the gene trap insertion showed that embryos with strong GFP expression had decreased levels of endogenous gene transcripts compared to non-GFP siblings (S1 Fig). For each gene, the transcript bands were quantified in ImageJ and normalized to *ß-actin*; the average percent decrease is shown Table 1. Taken together, these data support that the identified genes are the target of the gene trap disruptions, leading to GFP expression in hematopoietic cells.

### Identification of *agtpbp1* and *eps15L1* as genes important for T-cell development

**Fig 4:**
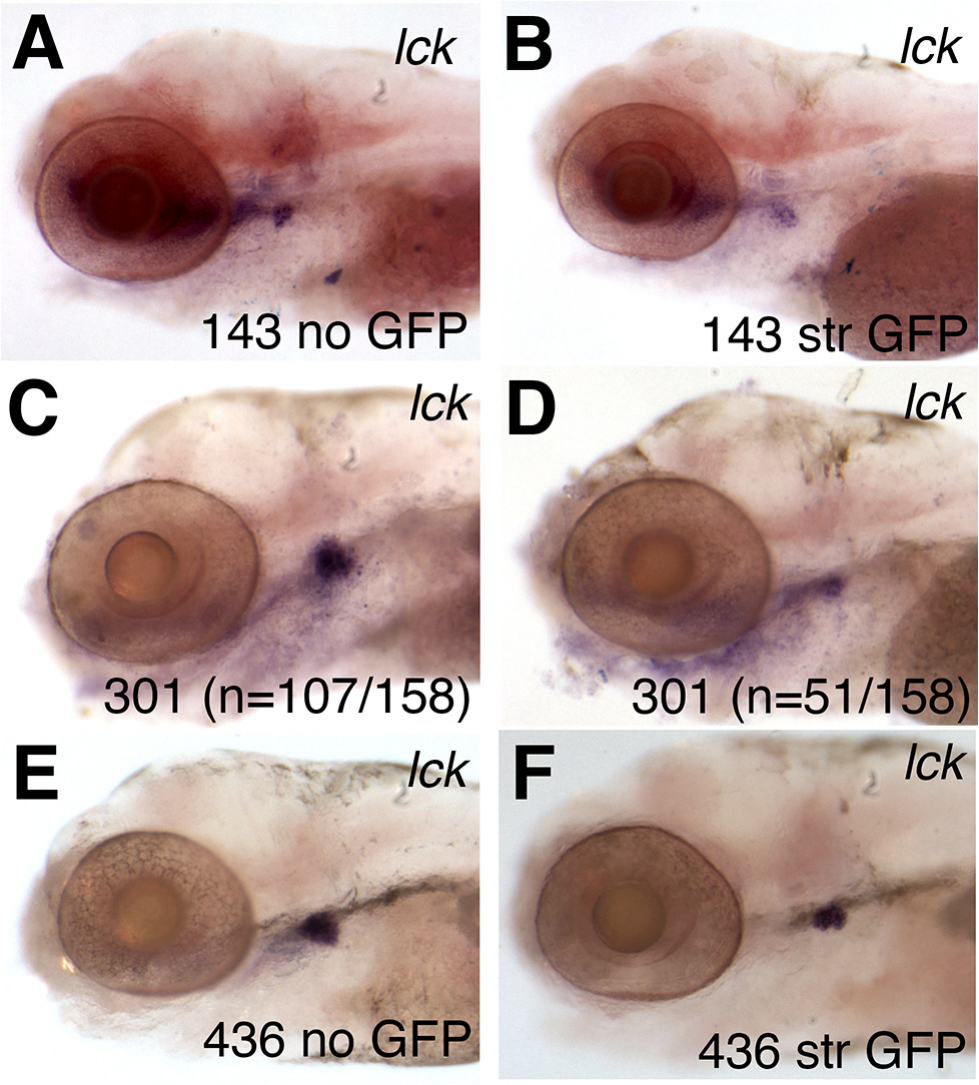
Decreased expression of lymphoid markers in gene-trap mutant embryos. (A-F) Whole mount RNA in situ hybridization (WISH) of *lck* in 5 dpf larvae from the lines *fcc143* (143) (A-B), *agtpbp1* ^*fcc301*^ (301) (C-D) and *eps15L1* ^*fcc436-P1*^ (436) (E-F). Representative embryos displaying no GFP (A, E) or normal expression of *lck* (C) are compared to siblings with strong (str) GFP (B, F) or low levels of *lck* expression (D). In line *agtpbp1* ^*fcc301*^, the GFP expression levels varied, making it difficult to distinguish heterozygous and homozygous carriers. The number of embryos that showed the representative phenotype is indicated in panels C and D.

Most of the lines with GFP expression in embryonic hematopoietic cells disrupt genes that have not previously been implicated in hematopoietic development. To address whether the disrupted genes are essential for hematopoietic development, we first used whole mount RNA in-situ hybridization (WISH) of cell-type specific markers to assess the effects of the gene mutation on the development of the following lineages. We assessed primitive myeloid cells marked by *mpx*, primitive erythroid cells expressing *hbae1-globin* and the definitive HSC/progenitor cell marker *c-myb* were examined in 2 dpf embryos. To evaluate effects on more differentiated definitive hematopoietic lineages, at 6 dpf we investigated development of T cells, myeloid cells, and erythrocytes by performing WISH using probes for *lck*, *mpx*, and *hbae1*, respectively. WISH was performed on F2 or F3 embryos generated from in-crossed heterozygous carriers of disrupted genes. Genotyping of individual embryos at these stages can be difficult. However, in most of the lines 25% of the larvae exhibited distinctly stronger GFP expression, suggesting that these embryos contained two copies of the disrupted gene. We did not detect any abnormalities in *cmyb-*, *mpx*- or *hbae1*-expressing cells (S5 Fig and S3 Table). However, 3 gene-trap lines showed clear reductions in *lck* marking of the thymus at 6 dpf (Fig 4), suggesting that T cell development was impaired. In two of the lines, *fcc143* (Fig 4A and 4B) and *eps15L1* ^*fcc436-P1*^ (Fig 4E and 4F), the decreased *lck* expression correlated with embryos that displayed very strong GFP expression, suggesting that these embryos were homozygous for the disrupted genes. In a third line, *agtpbp1* ^*fcc301*^ (Fig 4C and 4D), the strong versus weak GFP expression levels could not be easily discerned. Duplicate experiments examining progeny from incrosses of *agtpbp1* ^*fcc301*^ GFP^+^ carriers showed that an average of 32% (n = 158, 2 experiments) of the offspring showed weaker *lck* expression compared to GFP^-^ siblings. Next we visually assessed the GFP patterns in *fcc143*, *agtpbp1* ^*fcc301*^ and *eps15L1* ^*fcc436-P1*^ siblings that display strong versus weak GFP expression at 2 dpf, although line *fcc301* had a range of GFP expression levels. We did not detect differences in the GFP patterns between siblings albeit the cells showing stronger GFP were easier to detect (S6 Fig). This indicates that at this age homozygous gene-trap carriers do not display obvious defects in the generation of GFP marked cells.

To verify that the identified gene trap indeed results in the observed GFP expression pattern and mutant phenotype we evaluated the expression pattern and morpholino-mediated phenotype of the affected genes. Since the gene trap *gal4* and subsequent GFP expression rely on transcription from the endogenous gene promoter, GFP should reflect the normal expression pattern of the disrupted gene. We identified *ubap1* as a candidate target gene in line *fcc143* that displays GFP expression in thymic epithelium. However, WISH analysis of *ubap1* expression showed a largely non-overlapping pattern compared to the GFP expression in this line and morpholino knockdown of *ubap1* did not phenocopy the mutant (S7 Fig). It is likely that the insert responsible for the phenotype and GFP expression is closely linked to the *ubap1* insert, but affects a different locus.

**Fig 5:**
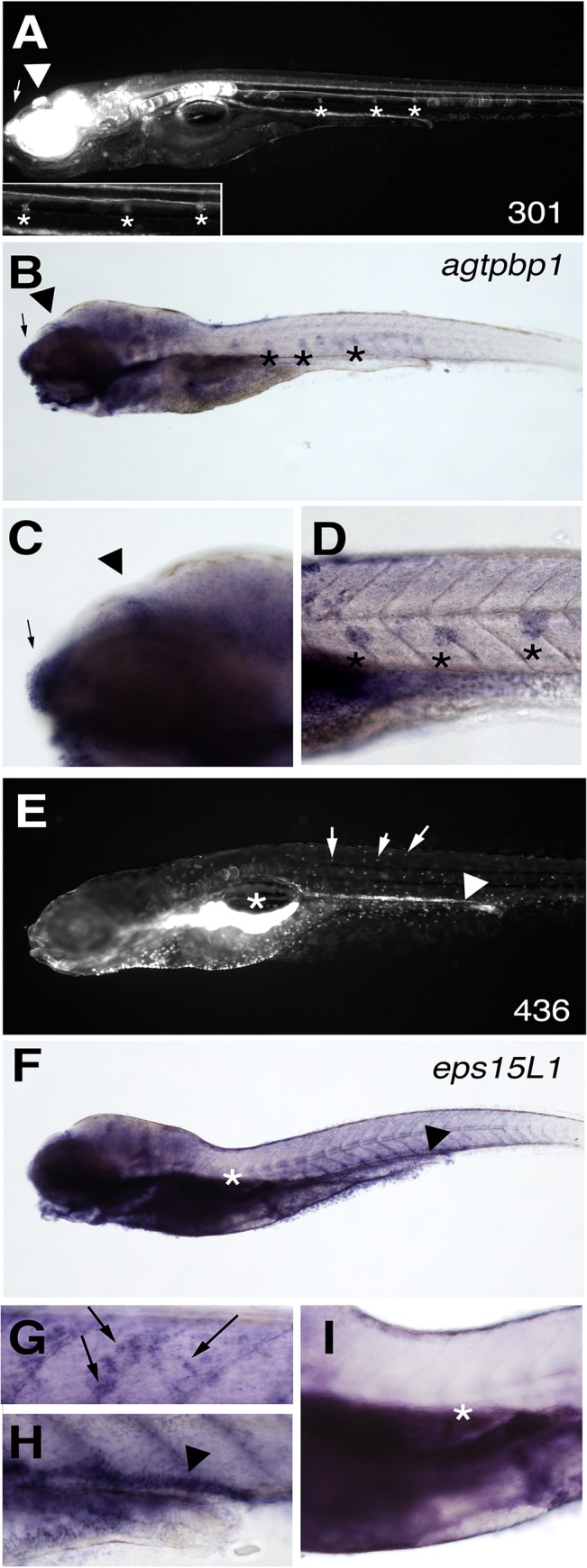
Endogenous gene expression matches the gene-trap GFP expression pattern for *agtpbp1* and *eps15L1* lines. (A) The GFP pattern in a representative 6 dpf embryo from the *agtpbp1* ^*fcc301*^ line. (B-D) WISH of *agtpbp1* at 6 dpf. Lateral line cells (*), epiphysis (arrowhead) and nasal pit (arrow) are indicated. (C-D) Magnified views of regions of the embryo shown in B. (E) The GFP pattern in a representative 6 dpf embryo from line *eps15L1* ^*fcc436*^. (F-I) WISH of *eps15L1* at 6 dpf. Pancreas (under *), kidney (arrowhead) and skin cells (arrows) are indicated. (G-I) Magnified views of regions of the embryo shown in F.

*Agtpbp1* ^*fcc301*^ gene trap carriers express GFP in the nose, eye, pituary gland, brain, notochord lateral line nerve, lateral line sensory organs and kidney (Fig 5A). WISH analysis of *agtpbp1* at 6 dpf shows expression in nose, eye, hypophysis, brain, and lateral line but we could not detect expression in the notochord, lateral line nerve or kidney (Fig 5B–5D). WISH analysis of *agtpbp1* at 2 dpf showed expression in the nose, eye, central nervous system and faintly in cells in the caudal hematopoietic region (S8A and S8B Fig), indicating that the GFP pattern is overlapping, although not a perfect reflection of endogenous gene expression. WISH analysis of *eps15L1* at 6 dpf showed that this gene is expressed in skin, kidney and pancreas, which overlaps exactly with the GFP expression pattern *fcc436* carriers (Fig 5E–5I). Expression of *eps15L1* at 2 dpf overlaps the GFP pattern in *fcc436* carriers at this age, and was observed in cells in the AGM and CHT regions, although RNA expression was detected throughout the embryo (S8C and S8D Fig). Possible explanations for why the GFP expression does not perfectly reflect endogenous gene expression include that the GFP protein is likely more stable than RNA transcripts, thus the GFP may be retained in cells from expression at earlier developmental stages, or that the level of sensitivity of the UAS-mediated detection system is higher than RNA in situ hybridization. Nonetheless, these data are consistent with *agtpbp1* and *eps15L1* being the gene trap targets in the *fcc301* and *fcc436-P1* lines, respectively.

**Fig 6:**
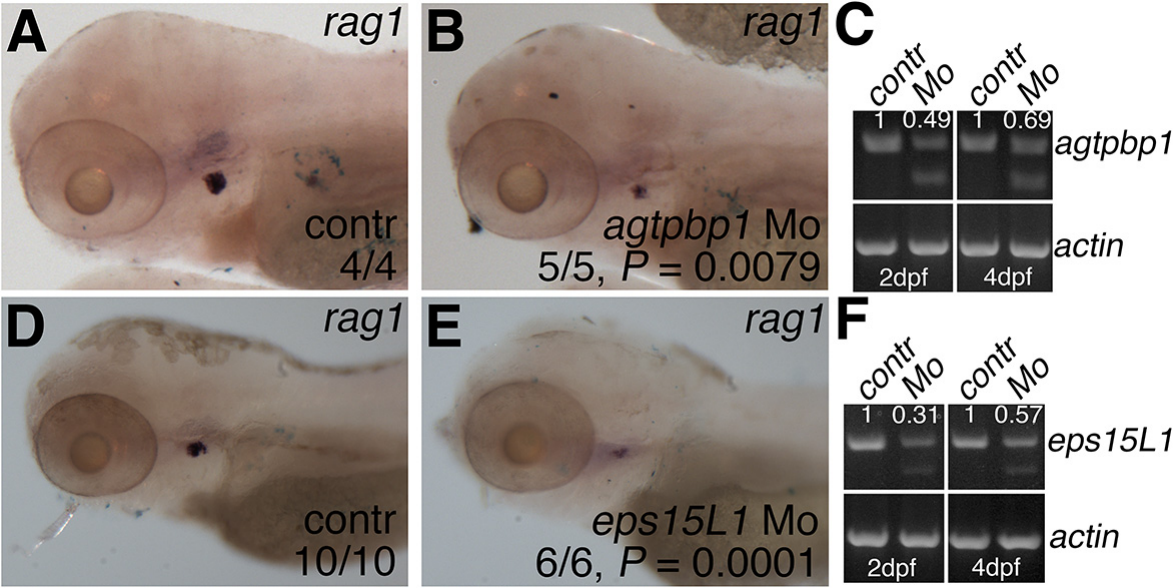
Morpholino knockdown of *agtpbp1* and *eps15L1* results in decreased lymphoid *rag1* expression. (A-B) WISH of *rag1* in 4 dpf control (A) and *agtpbp1* (B) morpholino-injected embryos. (C) RT-PCR of *agtpbp1* and *ß-actin* in pooled control or morphant embryo samples. Quantitation of the normal transcript band normalized to *ß-actin* is indicated. (D-E) WISH of *rag1* in 4 dpf control (A) and *eps15L1* (B) morpholino-injected embryos. (C) RT-PCR of *eps15L1* and *ß-actin* in pooled control or morphant embryo samples. Quantitation of the normal transcript band normalized to *ß-actin* is indicated. Quantitation is in arbitrary units (A.U.), and relative to wild-type level which is set at 1. Head region of the embryos is shown in lateral views, anterior to the left. P values were determined using Fisher’s exact test.

We utilized antisense morpholinos to determine if knocking down *agtpbp1* and *eps15L1* replicated the defects in T cell development that we observed in the mutants. Knockdown of *agtpbp1* severely disrupted early development of the larvae. Morpholino toxicity can cause non-specific *p53*-dependent cell death [32]. Co-injection of a *p53* morpholino with low doses of the *agtpbp1* morpholino suppressed the morpholino toxicity and resulted in morphologically normal larvae (Fig 6A and 6B). In all subsequent experiments, *agtpbp1* morphants and controls were co-injected with *p53* morpholino. RT-PCR analysis showed that the *agtpbp1* morpholino at this low dose decreased endogenous *agtbp1* transcripts by 51% on day 2 and 31% on day 4 (Fig 6C). WISH of *rag1* showed that 4 dpf morpholino-injected embryos (morphants) had reduced thymic expression compared to uninjected controls or *p53* morphants (Fig 6A and 6B), suggesting that partial depletion of *agtpbp1* disrupts T cell development. *Agtpbp1* morphants continued to display decreased *rag1* expression at 5 dpf (S9A Fig). Consistent with this finding, we acquired images of *rag2*:mCherry transgene expression in the thymus of *agtpbp1* morphants and control embryos at 5 dpf (S10 Fig). ImageJ quantification of the mCherry expression showed that the morphants had significantly decreased levels of thymic mCherry (S9B Fig, P = 0.0056). Thus, *agtpbp1* deficiency impairs T cell development.

To better understand how *agtpbp1* deficiency disrupts lymphopoiesis, we evaluated development of the *foxn1* ^+^ thymic epithelium and *ikaros*-expressing lymphoid progenitors in *agtpbp1* ^*fcc301*^ mutants. WISH analysis showed that *agtpbp1* ^fcc301^ carriers and GFP- siblings displayed indistinguishable patterns of thymic *foxn1* and *ikaros* expression at 5 dpf and 3 dpf, respectively (S11A and S11B Fig). This indicates that the *agtpbp1* deficiency-induced defect in lymphopoiesis occurs despite normal development of the thymic epithelium, an important component of the thymus microenvironment, and normal seeding of the thymus by *ikaros*-positive lymphoid progenitors.

Morpholino-mediated knockdown of *eps15L1* did not affect the morphology of the developing embryo (Fig 6D and 6E). RT-PCR analysis showed that endogenous transcripts were decreased in morphants by 69% on day 2 and 43% on day 4 to control embryos (Fig 6F). Thus, both the GBT-B4 insertion and morpholino-mediated targeting of *eps15L1* result in a partial silencing of the gene. Examining *rag1*-expression by WISH at 4 dpf and 5 dpf showed a strong reduction in morphants compared to control embryos (Fig 6D and 6E and S9C Fig). We next assessed *rag2*:*mCherry* transgene expression in *eps15L1* ^*fcc436-P1*^ mutants and *eps15L1* morphants. F0 fish were bred with *Tg(rag2*:*mCherry)* transgenic fish and F1 GFP^+^/RFP^+^ embryos were grown to adulthood. F1 double heterozygous carriers were backcrossed with the gene trap line and mCherry expression analyzed in homozygous (strong GFP) carriers or wild-type (no GFP) siblings. Images were acquired of the thymic mCherry expression (S10 Fig. ImageJ was used to quantify thymic mCherry expression (S9 D Fig). Mutant embryos with strong GFP expression had significantly decreased levels of thymus mCherry (*P* = 0.0002). Similarly, *eps15L1* morphants had significantly decreased levels of thymus mCherry compared to controls (S9E Fig, *P* = 0.0003). These data confirm that depletion of *eps15L1* disrupts lymphoid development.

We used WISH to examine whether the development of *foxn1* ^+^ epithelium and the *ikaros* ^+^ progenitor population in the thymus were defective in *eps15L1* ^*fcc436-P1*^ mutants. Similar to *agtpbp1* ^*fcc301*^ mutants, we found that *eps15L1* ^*fcc436-P1*^ mutants (GFP high) and siblings (GFP low and negative) displayed identical patterns of thymic *foxn1* and *ikaros* expression at 5 dpf and 3 dpf, respectively (S11C and S11D Fig). Together, these data indicate that *agtpbp1* and *eps15L1* are important for the development of lymphoid progenitors within the thymus.

The amino acid sequences of Atgpbp1 and Eps15L1 are highly conserved between zebrafish, mouse and human proteins, suggesting that the mammalian counterparts of these genes are likely to have similar functions (S12 Fig). Using the Immunological Genome Project (ImmGen) data browser (gene skyline), we examined expression of the murine homologs of *Agtpbp1* and Eps15L1 in purified stem/progenitors, B lineage cells and T lineage cells [49]. *Agtpbp1* had the highest expression levels in germinal center and follicular B cells from the spleen and T cell double positive (CD4^+^8^+^) blasts from the thymus compared to other T cell subsets (S13 Fig). The alpha-beta T cell lineage gives rise to double positive blasts that are poised to become CD4 or CD8 cells or to die. *Eps15L1* expression levels are similar between stem/progenitors, B lineage cells and T lineage cells, with slightly increased levels in pre-T, double negative cells (DN2B and 3A fractions) from the thymus (S13 Fig). DN2/3 is the stage when gamma-delta versus alpha-beta T cell lineages are specified. The expression of the murine homologs is consistent with genes that are important for T cell development in the thymus of vertebrates. In sum, our data reveals a previously unexplored role for *agtpbp1* and *eps15L1* in T cell development in zebrafish and establishes a series of lines that will likely be useful tools in understanding the role of these genes in embryonic and adult hematopoiesis.

## Discussion

Here we report a small-scale genetic screen to discover genes important in hematopoietic development. Our distinct approach used a newly engineered transposon vector, which marked targeted cells and enabled flow cytometric analysis, streamlined the screening process and identified many novel genes, two in particular that play important roles in T cell development. This approach has multiple benefits: (1) easy and non-toxic generation of mutant fish, (2) ready identification of genes specifically expressed in the cells of interest, (3) facilitates identification of homozygous mutants by the intensity of the GFP marker proteins, and (4) allows easier and more rapid identification of the disrupted gene relative to chemical mutagenesis. We found that the insertions reduce, but do not necessarily eliminate normal (wildtype) transcripts. Furthermore, there is the possibility of a bias for the transposon to be inserted in regions containing actively transcribed genes during mutagenesis [50]. Nonetheless, we identified insertions in a functionally diverse set of genes, including those whose products are involved in cytoskeleton formation, vesicle transport and transcriptional regulation, which gave rise to a broad spectrum of GFP expression patterns.

As a unique aspect of our approach, the transposon-based GFP labeling allowed us to use flow cytometry to purify marked cells from young adult fish and test whether the population contained cells expressing T and/or B cell specific markers. We identified 18 lines in which *igH-*μ and/or *lck* expression was detected in the purified GFP^+^ populations, indicating the presence of B or T cells, respectively. The expression analysis indicated that some genes showed specificity to a particular type of lymphoid cell, while other lines showed GFP expression in both *igH-*μ and *lck*-expressing cells. These data indicate that the disrupted genes have cell-type specific expression during lymphopoiesis, some of which may be useful markers in evaluating adult lymphoid populations. Future studies can utilize these lines (1) to mark subsets of lymphoid cells and (2) test if homozygous mutants affect T and/or B cell development in zebrafish embryos or young adults.

We used multiple methods to identify the transposon insertion responsible for GFP expression and mutant phenotypes. To confirm the disrupted gene, we assessed 6 GFP positive and 6 GFP negative larvae for linkage of GFP expression to the identified genomic insertion. This genetically maps the insertion to less than an 8.3 cM interval that is linked to the GFP expression-inducing insertion, providing a strong, but not absolute, correlation of the identified insertion with the GFP expression. To further confirm the gene disruption in the lines showing defects in T cell development, we used two approaches: (1) we used morpholinos to phenocopy the mutants and (2) examined if endogenous gene expression pattern resembled the GFP pattern. This approach confirmed identification of the gene trap sites for lines *agtpbp1* ^*fcc301Gt*^ and *eps15L1* ^*fcc436Gt*^. However, we were unable to phenocopy mutants from line *fcc143* using a morpholino, suggesting the possibility of insurmountable morpholino toxicity, off target effects or that an additional transposon that is linked to the GFP expression is responsible for the lymphoid defects displayed by *fcc143* mutants. Additional future analysis is necessary to identify the underlying cause of the lymphoid defect in this line.

Our efforts showed that deficiency of *agtpbp1* and *eps15L1* inhibit T cell development. The role of these genes in hematopoiesis is previously unexplored. In zebrafish embryos deficient for either gene, the development of the thymic epithelium and presence of lymphoid progenitors in the thymus appears normal. This suggests that *agtpbp1* and *eps15L1* deficiency disrupts the development of lymphoid progenitors within the thymus.

The gene encoding ATP/GTP binding protein 1 (*agtpbp1*/*nna1/ccp1*) was initially cloned from murine spinal cord cells and contains a zinc carboxypeptidase domain and an ATP/GTP-binding motif [51]. AGTPBP1 acts to shorten polyglutamate side chains on Tubulin and other proteins [52]. Previous analysis in mice carrying mutations in *agtpbp1* revealed defects in a number of different cell types, including Purkinje, retinal photoreceptors and olfactory bulb mitral cells [53–56], and the lengthening of the polyglutamate side chains on Tubulin was linked to neuronal degeneration [52], but no thymic defects were described. We found that zebrafish *agtpbp1* was expressed in neural cells and other tissues, consistent with previous reports. We also observed low numbers of circulating GFP^+^ cells in embryo carriers and the mutant and morphant embryos displayed defects in T cell development. However, the GFP^+^ population purified from young adult carriers did not express *igH-*μ and *lck*. Possible explanations include that *agtpbp1* functions in lymphoid progenitors, prior to the developmental window when *igH-*μ and *lck* are expressed. Consistent with this idea, the *agtpbp1* gene-trap line showed GFP expression in the AGM region but robust expression in the thymus was not observed. It is possible that *agtpbp1* is repressed in mature T cells but retained in other lineages. Alternately, albeit less likely, the transposon may tag different hematopoietic populations at these two ages or ablation of *agtpbp1* may result in non-cell autonomous defects in T cell development. Nonetheless, our results support a previously unrecognized role for *agtpbp1* in regulating lymphoid development.

Epidermal growth factor receptor pathway substrate 15-like 1 (*EPS15L1*/*EPS15R*) is required for clathrin-dependent endocytosis, and mediates internalization of the EGF and Transferrin receptors [57]. Eps15L1 is also detected in the nucleus [58] and facilitates the transcription of BMP-responsive genes, and interacts with Smad1, in *Xenopus* embryos [59]. The regulation of Smad1 is important in the emergence of hematopoietic stem cells in zebrafish [60], and the Smad pathway regulates B cell function and T cell development [61]. An epidemiological study correlated genomic markers with white blood cells counts of participants and linked a SNP within the *EPS15L1* locus to lymphocyte counts [62], suggesting that this gene may be important in human lymphopoiesis. Zebrafish *eps15L1* gene-trap carriers displayed GFP^+^ cells in the AGM region but GFP expression in the thymus was not detected. However, the GFP^+^ cells from young adults expressed *igH-*μ and *lck*. This discrepancy may be due to our inability to visually detect low levels of GFP expression or low numbers of GFP^+^ cells in individual fish compared to the highly sensitive flow cytometric analysis of cells from pools of embryos. Alternately, the gene-trap may mark different hematopoietic populations at these two ages. In zebrafish embryos, *eps15L1* deficiency results in decreased *rag*-expressing cells in the thymus despite normal epithelial development and infiltration by progenitors. Although we have not determined how *eps15L1* deficiency impacts adult lymphopoiesis, our results clearly establish an important role for *eps15L1* in lymphoid development in zebrafish embryos.

In sum, using forward genetics we have identified two previously unrecognized genes that are important in immune cell development. We identified 12 lines in which embryonic hematopoietic tissues are marked by GFP and 18 lines in which the GFP^+^ cells in young adults express T and/or B cell markers. These lines represent unique tools as they label subsets of hematopoietic cells and, thus, can facilitate hematopoietic analyses, especially in the study of immunity in young adult fish. Moreover, within a given line, the *GBT-B4* insertion can be commandeered as a tissue-specific Gal4 driver for UAS-driven expression of cancer/immunity modifiers. In sum, these studies establish a previously unrecognized role for the *eps15L1* and *agtpbp1* genes in T cell development, and developed a profusion of new reagents to aid future in vivo studies of blood cell development and immune function.

## Supporting Information

S1 Fig Full-length images of gels. (A-B) PCR linkage analysis with genomic DNA from individual embryos. Linkage to the indicated genes were tested using control and gene-specific primers in embryos showing the indicated GFP expression level. Gsp = gene specific primers; con = control primers. (C) RT-PCR linkage analysis with cDNA from pools of GFP-positive and pools of GFP-negative embryos for the indicated genes. (D-E) RT-PCR analysis of gene-trap target expression level in pools of GFP-negative/GFP-low (WT) versus GFP-high (mut/m) embryos from the indicated lines/genes. (F) RT-PCR analysis of morpholino target gene expression in pools of control and morphant embryos as indicated.
(TIF)

S2 Fig Linkage of transposon insertion in lines fcc 301, 436 and 667 to GFP expression. (A) RT-PCR detection of fusion transcripts of *agtpbp1* fusion with *gal4* (top left) or *eps15L1* fusion with *gal4* (bottom left) in individual GFP^+^ embryos or 2 pools of 3 GFP^-^ embryos. Expression of *ß-actin* was examined in GFP^-^ samples to confirm sample integrity (right panels). The gene-specific fusion band is indicated by an arrowhead. (B) PCR detection of genomic insertion (*abi1a*-insertion/ins) and control for DNA integrity (*glis1b*) in individual GFP^+^ and GFP^-^ embryos. Note the positive bands in GFP^+^ samples and absence of the genotyping band in GFP^-^ samples.
(TIF)

S3 Fig Images of RT-PCR gels from expression analysis of GFP^+^ purified cells. (A) The gel image from Fig 3B. (B) The image used to quantify expression of the indicated genes, which were normalized to actin to determine expression levels. (C) The uninverted image of the composite gel Fig. Sections that originated from different gels are indicated. (D) Gel images from which the composite Fig. was generated. The gel sections used for the final Fig. are indicated.
(TIF)

S4 Fig Confocal analysis of the localization of gene-trap GFP^+^ cells compared to transgenic (tg) *rag2*:mCherry lymphoid cells. (A) GFP^+^ cells in the thymus of 6 dpf line *fcc143* embryos are distinct from, and surround, *rag2*:mCherry lymphoid cells. (B) Coexpression of line *fcc337* GFP with transgene *rag2*:mCherry in a thymus of 6 dpf embryos. Yellow color = coexpression.
(TIF)

S5 Fig Normal emergence of definitive progenitor cells and primitive myeloid and erythroid cells in *agtpbp1* ^fcc301Gt^ */Tg(GBT-B4)fcc301* and *eps15L1* ^fcc436-P1Gt^ */Tg(GBT-B4)fcc436-P1* mutant embryos. (A) WISH of *cmyb* in 2 dpf embryos siblings from lines *fcc* 301 (top) and 436 (middle and bottom). There was no difference in the *cmyb* patterns between siblings. (B) WISH of *mpx* and *hbae1* (in red) in 2 dpf embryos siblings from lines *fcc* 301 (top) and 436 (middle and bottom). There was no difference in the expression patterns between siblings. The number of siblings that display the representative phenotype is indicated in the panels.
(TIF)

S6 Fig Comparison of GFP expression levels in fcc143, *agtpbp1* and *eps15L1* gene-trap lines. Images of representative 2 dpf siblings displaying low and high levels of GFP were acquired using the same exposure parameters for a given magnification.
(TIF)

S7 Fig Evidence that *ubap1* is not the gene-trap target in line fcc143. (A) RT-PCR analysis of *ubap1* and *actin* expression in control and *ubap1* morphants co-injected with *p53* morpholino. (B) Brightfield images of groups of control and *ubap1* morphants. *Ubap1* morphants display widespread developmental defects, unlike fcc143 GFP-high embryos (see Fig 4B). (C) WISH of *ubap1* antisense and sense probes in 2 and 6 dpf embryos. *Ubap1* expression, shown by the antisense probe, was not detected in the caudal hematopoietic tissue or thymus in contrast to the GFP pattern in fcc143 carriers (see Fig 2).
(TIF)

S8 Fig Whole mount expression analysis of *agtpbp1* and *eps15L1*. (A-D) WISH of the indicated genes in 2 dpf embryos. (A) *Agtpbp1* expression is shown in purple. Image shows a lateral view of a representative embryo, anterior facing left. Embryo was deyolked. The boxed area is enlarged in the lower panel. Black arrows indicate *agtpbp1*-expressing cells in the caudal hematopoietic tissue (CHT). (B) Transverse sections through the trunk (top panel) and tail (bottom panel) regions to show *agtpbp1* expression in a 2-dpf embryo. Black arrows in the lower panel indicate positive cells in the CHT. (C) *Eps15L1* expression is shown in purple. Image shows a lateral view of a representative embryo, anterior facing left. Embryo was deyolked. The boxed area is enlarged in the lower panel. Black arrows indicate positive cells in the AGM and CHT. (D) Transverse sections through the trunk (top panel) and tail (bottom panel) regions to show *eps15L1* WISH analysis in a 2-dpf embryo. Black arrows indicate positive cells in the ventral wall of the dorsal aorta (da; AGM region) and CHT in the top and bottom panels, respectively. sp = spinal cord, no = notochord, da = dorsal aorta, cht = caudal hematopoietic tissue region.
(TIF)

S9 Fig Deficiency for *agtpbp1* and *eps15L1* inhibits lymphoid development. (A) WISH of *rag1* in 5 dpf control and *agtpbp1* morphants co-injected with *p53* morpholino. N is indicated. Images show lateral views of the left side of the head. Arrows indicate *rag1*-expressing cells in the thymus. (B) Levels of *rag2*:mCherry transgene expression in the thymus of 5 dpf control and *agtpbp1* morphants. Images of mCherry (B,D,E) in siblings were acquired using identical exposure settings. Fiji was used to quantify the whole mount expression from the acquired images (shown in S10 Fig). (C) WISH of *rag1* in 5 dpf control and *eps15L1* morphants. N is indicated. Two independent experiments were performed. Images show lateral views of the left side of the head. Arrows indicate *rag1*-expressing cells in the thymus. (D-E) Quantitation of the *rag2*:mCherry transgene expression in the thymus in lines *eps15L1* ^*fcc436-P1*^ (436) siblings at 6dpf (D) and control and *eps15L1* morphants at 5 dpf (E). P values for mCherry quantitation were determined using two-tailed Student’s T-test; P values for WISH were determined using Fisher’s exact test.
(TIF)

S10 Fig Images of mCherry expression in control compared to *ep15L1* and *agtpbp1* deficient embryos. Images of the thymus in individual siblings in an experimental set are shown. Images of control and gene deficient embryos were acquired using identical exposure parameters. Each thymus image represents a different embryo. The embryos and stages are indicated.
(TIF)

S11 Fig *Agtpbp1* and *eps15L1* mutants display normal thymic *foxn1* and *ikaros* expression patterns. (A) WISH of *foxn1* in 5 dpf *agtpbp1* ^*fcc301*^ siblings sorted prior to fixation by their level of GFP expression, although there was a range of GFP expression levels in this line. (B) WISH of *ikaros* in 3 dpf *agtpbp1* ^*fcc301*^ siblings separated prior to fixation based on their GFP expression level. (C) WISH of *foxn1* in 5 dpf *eps15L11* ^*fcc436-P1*^ siblings displaying the indicated GFP expression level. (D) WISH of *ikaros* in 3 dpf *eps15L11* ^*fcc436-P1*^ siblings sorted prior to fixation by their level of GFP expression. Orientation, GFP expression levels and N are indicated. Neg = negative. Black arrows/arrowheads indicate WISH^+^ cells in the thymus.
(TIF)

S12 Fig Conservation of Agtpbp1 and Eps15L1 protein sequences in vertebrates. (A) Alignment of amino acid sequence of Agtpbp1 from *Danio rerio*, *Homo sapiens* and *Mus musculus*. (B) Alignment of amino acid sequence of Eps15L1 from *Danio rerio*, *Homo sapiens* and *Mus musculus*. (A-B) Clustal analysis; identical amino acids are highlighted; accession numbers of the proteins are listed, zebrafish proteins are predicted. Clustal Format alignment was generated through the www.phylogeny.fr site.
(TIF)

S13 Fig ImmGen analysis shows expression of murine *Agtpbp1* and *Eps15L1* in purified hematopoietic populations. Gene skyline generated expression profiles of *Agtpbp1* and *Eps15L1* in purified hematopoietic populations as indicated. ImmGen = Immunological Genome Project, http://www.immgen.org/.
(TIF)

S1 Table Primer and morpholino sequences. The fcc line number, target gene, sequence of the oligomer and experimental use are indicated.
(PDF)

S2 Table Results from the screen including embryonic GFP expression, disrupted gene and insert location and results of RT-PCR analysis of GFP^+^ cells from 6 week old juvenile fish. (PDF)

S3 Table Results from whole mount RNA in situ analysis of hematopoiesis in gene-trap lines with embryonic marking of blood cells. (PDF)
