# Supplementary figures and images for: Mutagenesis Screen Identifies agtpbp1 and eps15L1 as Essential for T lymphocyte Development in Zebrafish
Source: PLoS One. 2015 Jul 10;10(7):e0131908. doi: 10.1371/journal.pone.0131908 (PMC4498767; doi:10.1371/journal.pone.0131908)

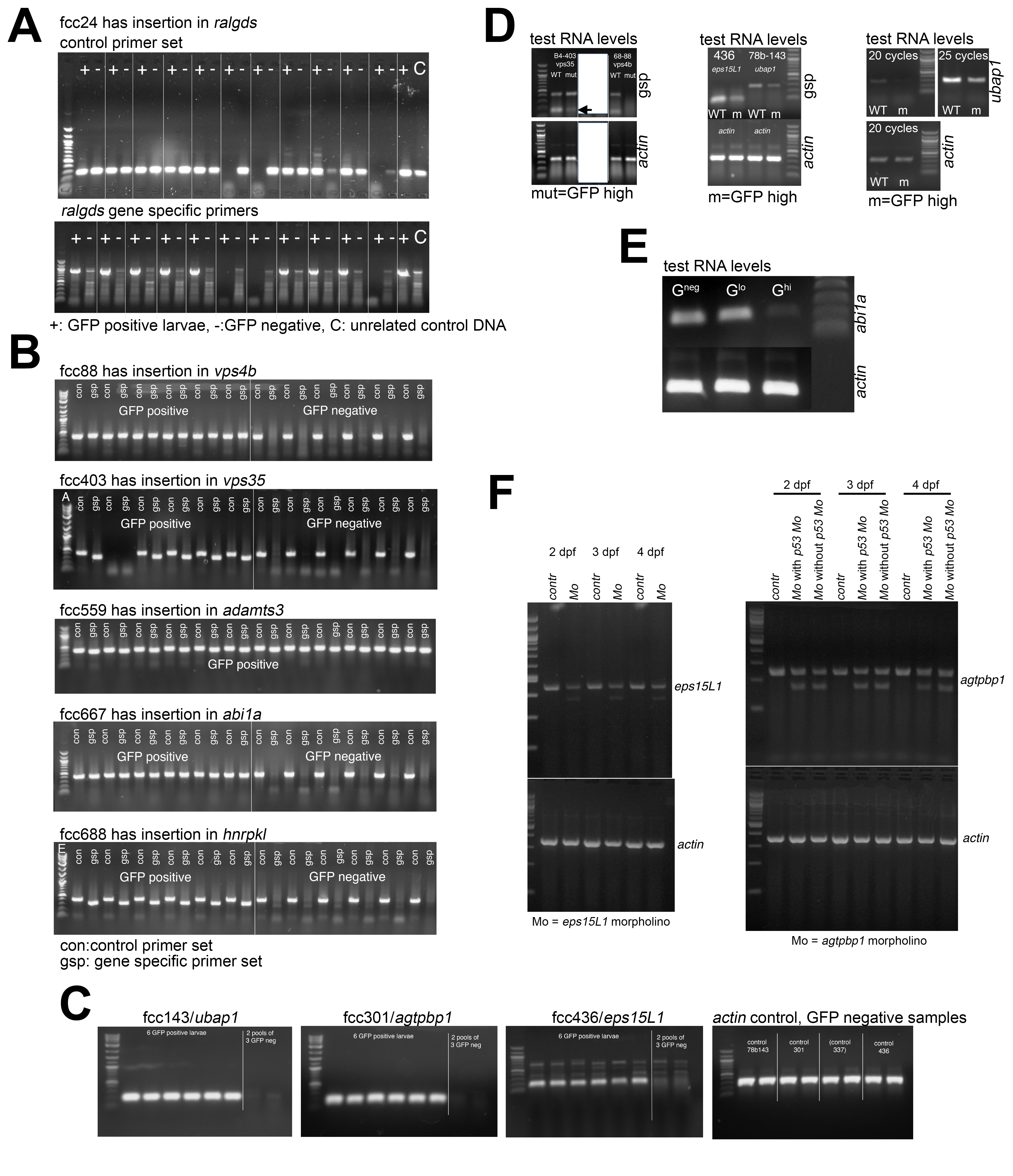

Supplement: S1 Fig — (A-B) PCR linkage analysis with genomic DNA from individual embryos. Linkage to the indicated genes were tested using control and gene-specific primers in embryos showing the indicated GFP expression level. Gsp = gene specific primers; con = control primers. (C) RT-PCR linkage analysis with cDNA from pools of GFP-positive and pools of GFP-negative embryos for the indicated genes. (D-E) RT-PCR analysis of gene-trap target expression level in pools of GFP-negative/GFP-low (WT) versus GFP-high (mut/m) embryos from the indicated lines/genes. (F) RT-PCR analysis of morpholino target gene expression in pools of control and morphant embryos as indicated. (TIF) [file pone.0131908.s001.tif]

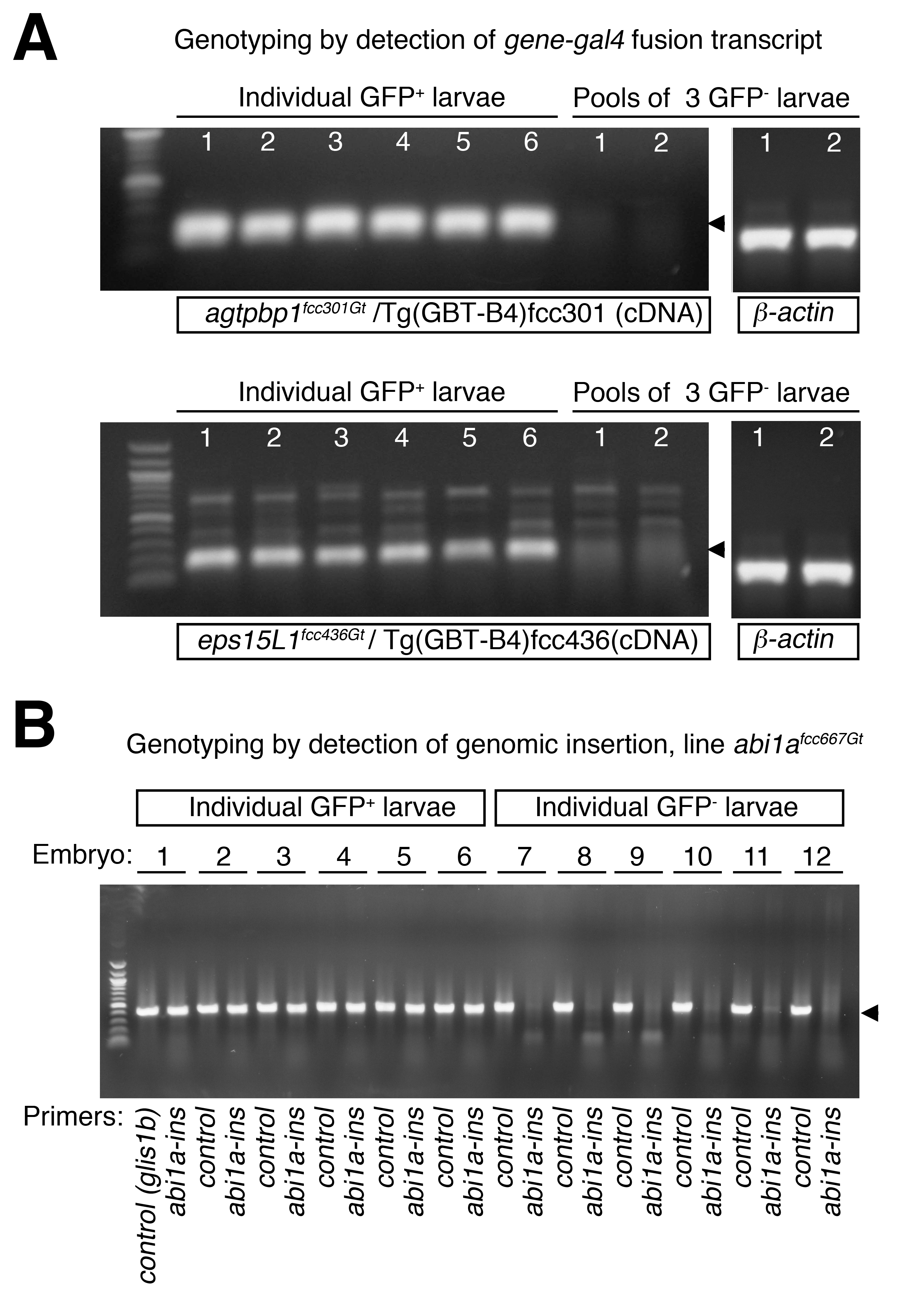

Supplement: S2 Fig — (A) RT-PCR detection of fusion transcripts of agtpbp1 fusion with gal4 (top left) or eps15L1 fusion with gal4 (bottom left) in individual GFP+ embryos or 2 pools of 3 GFP- embryos. Expression of ß-actin was examined in GFP- samples to confirm sample integrity (right panels). The gene-specific fusion band is indicated by an arrowhead. (B) PCR detection of genomic insertion (abi1a-insertion/ins) and control for DNA integrity (glis1b) in individual GFP+ and GFP- embryos. Note the positive bands in GFP+ samples and absence of the genotyping band in GFP- samples. (TIF) [file pone.0131908.s002.tif]

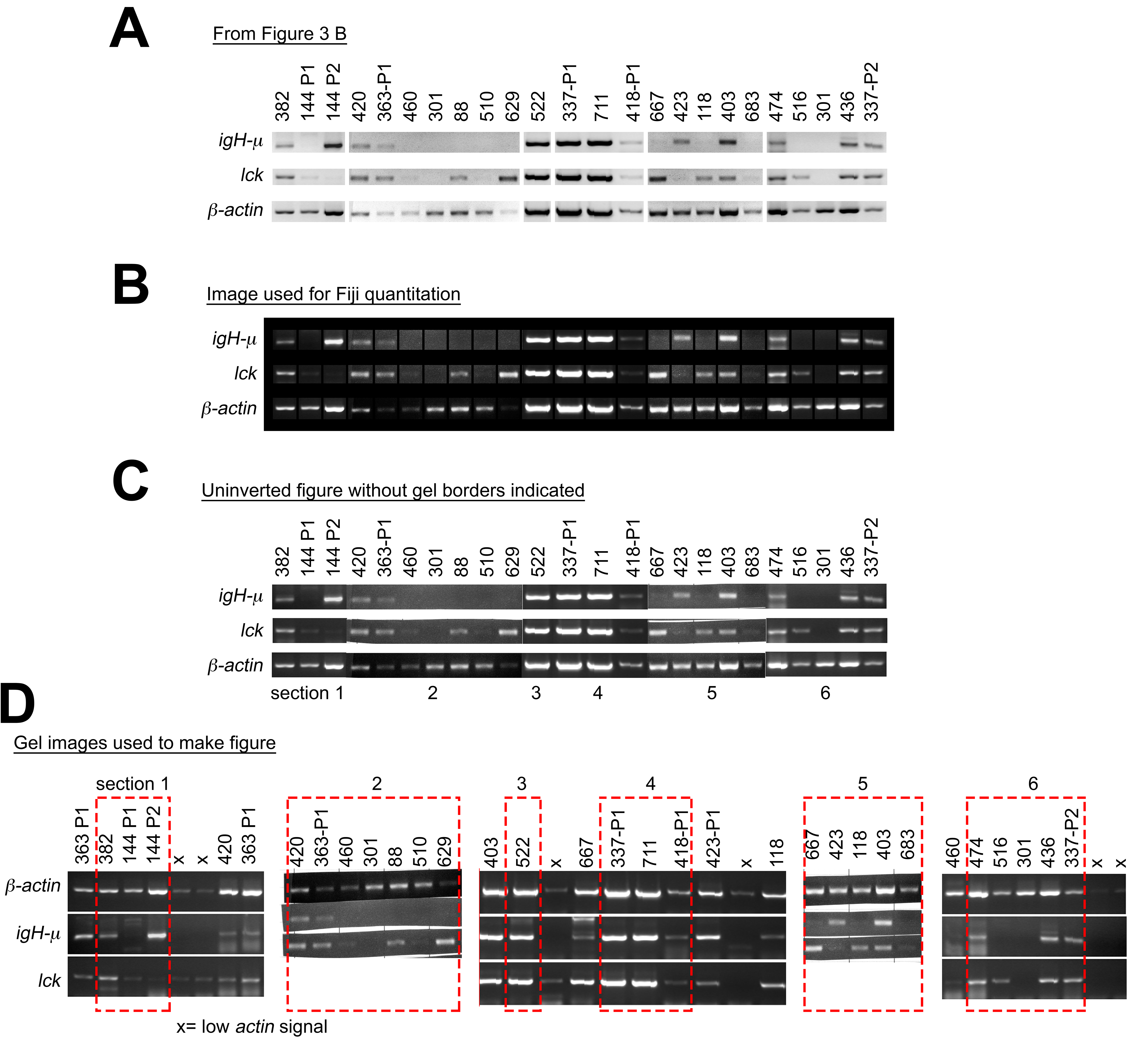

Supplement: S3 Fig — (A) The gel image from Fig 3B. (B) The image used to quantify expression of the indicated genes, which were normalized to actin to determine expression levels. (C) The uninverted image of the composite gel Fig. Sections that originated from different gels are indicated. (D) Gel images from which the composite Fig. was generated. The gel sections used for the final Fig. are indicated. (TIF) [file pone.0131908.s003.tif]

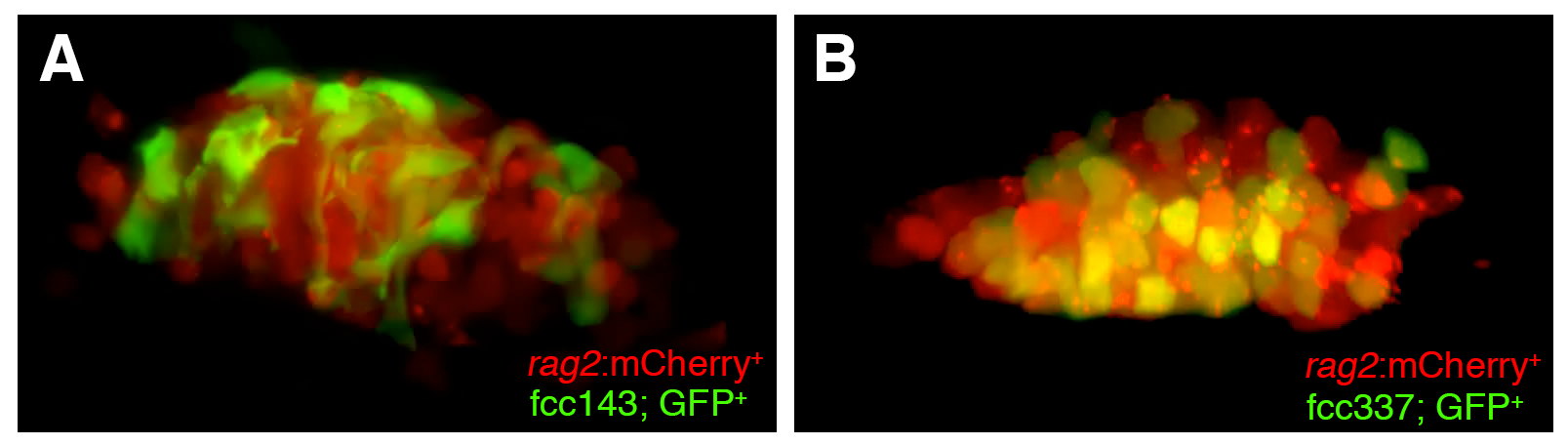

Supplement: S4 Fig — (A) GFP+ cells in the thymus of 6 dpf line fcc143 embryos are distinct from, and surround, rag2:mCherry lymphoid cells. (B) Coexpression of line fcc337 GFP with transgene rag2:mCherry in a thymus of 6 dpf embryos. Yellow color = coexpression. (TIF) [file pone.0131908.s004.tif]

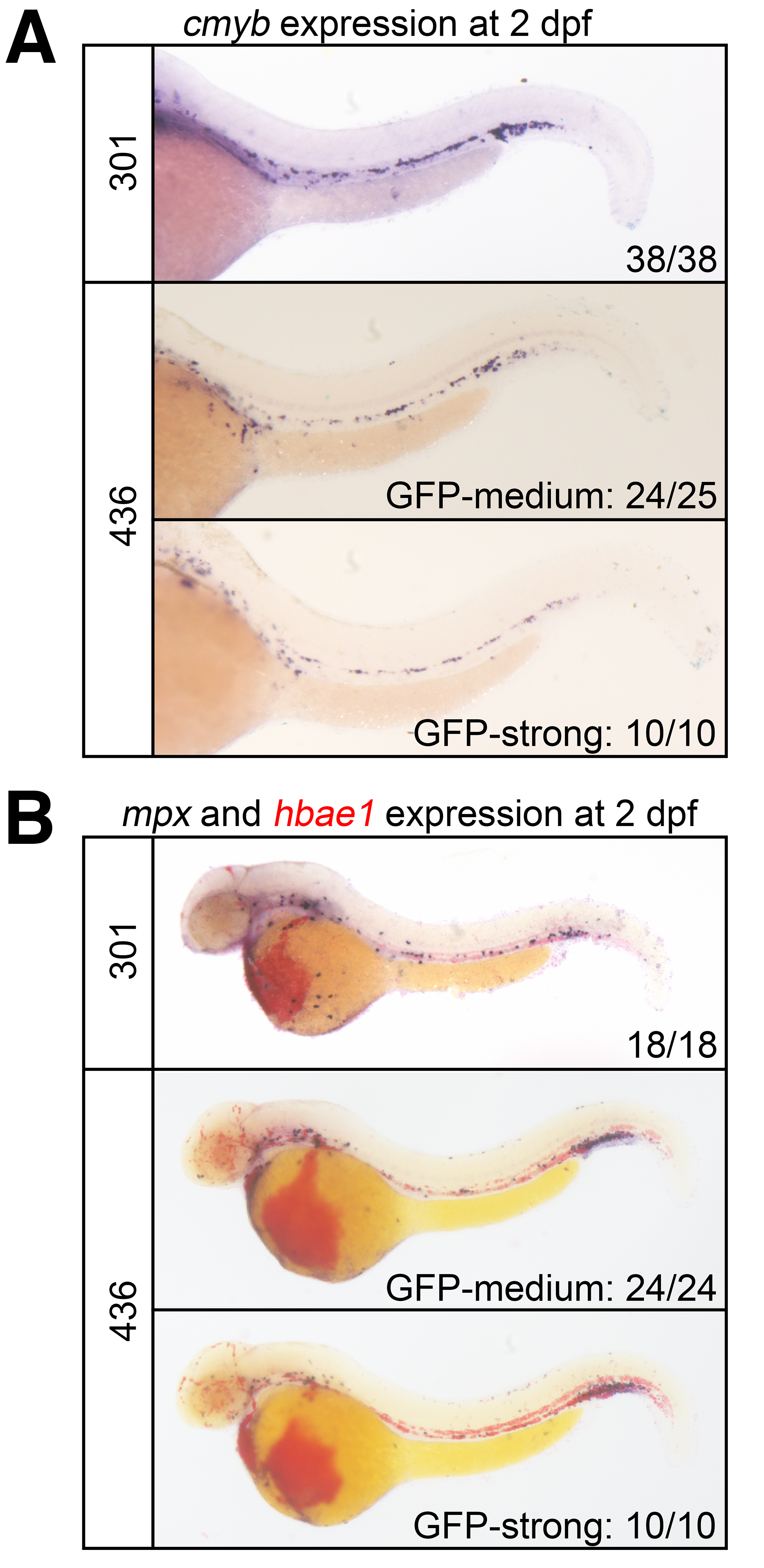

Supplement: S5 Fig — (A) WISH of cmyb in 2 dpf embryos siblings from lines fcc 301 (top) and 436 (middle and bottom). There was no difference in the cmyb patterns between siblings. (B) WISH of mpx and hbae1 (in red) in 2 dpf embryos siblings from lines fcc 301 (top) and 436 (middle and bottom). There was no difference in the expression patterns between siblings. The number of siblings that display the representative phenotype is indicated in the panels. (TIF) [file pone.0131908.s005.tif]

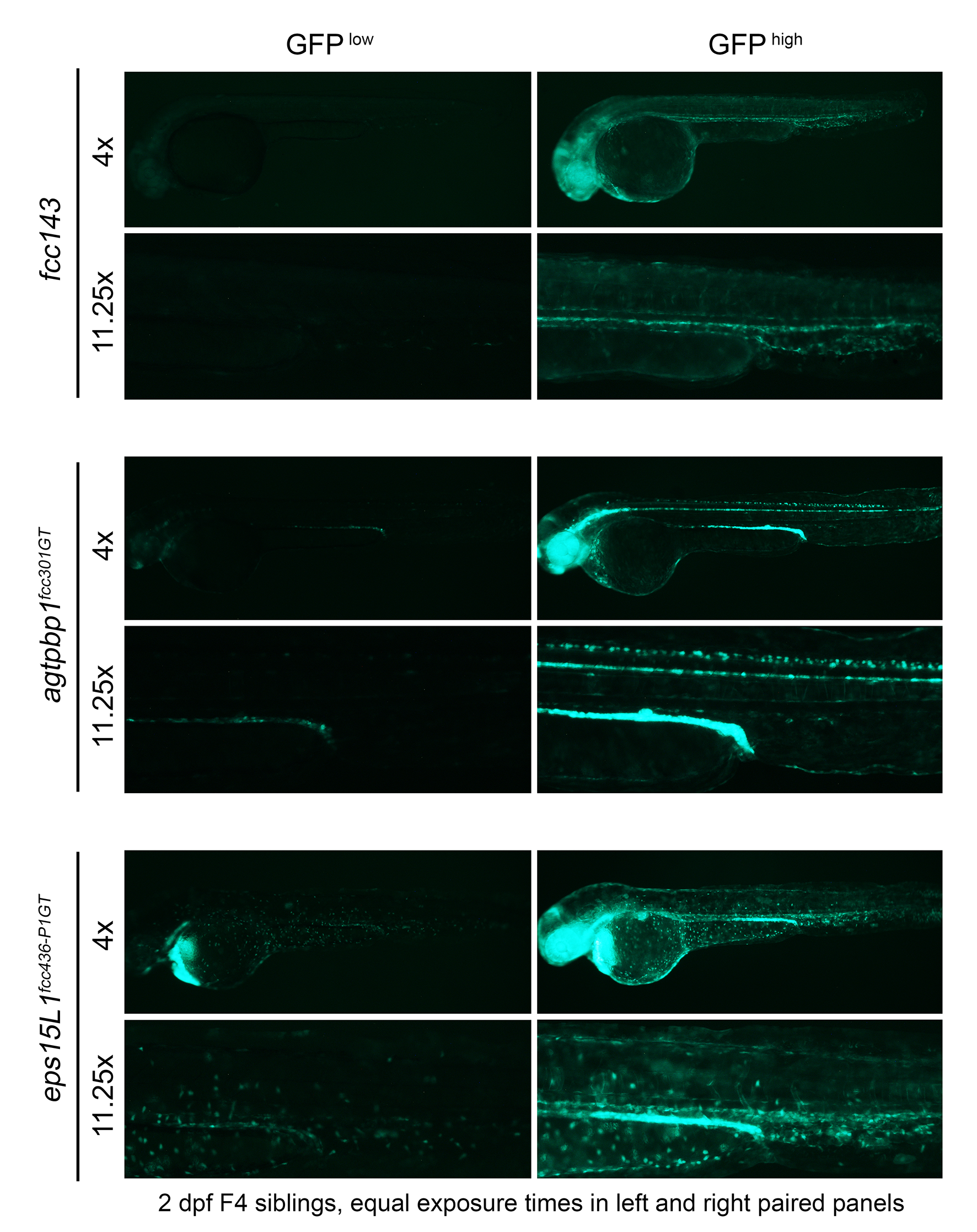

Supplement: S6 Fig — Images of representative 2 dpf siblings displaying low and high levels of GFP were acquired using the same exposure parameters for a given magnification. (TIF) [file pone.0131908.s006.tif]

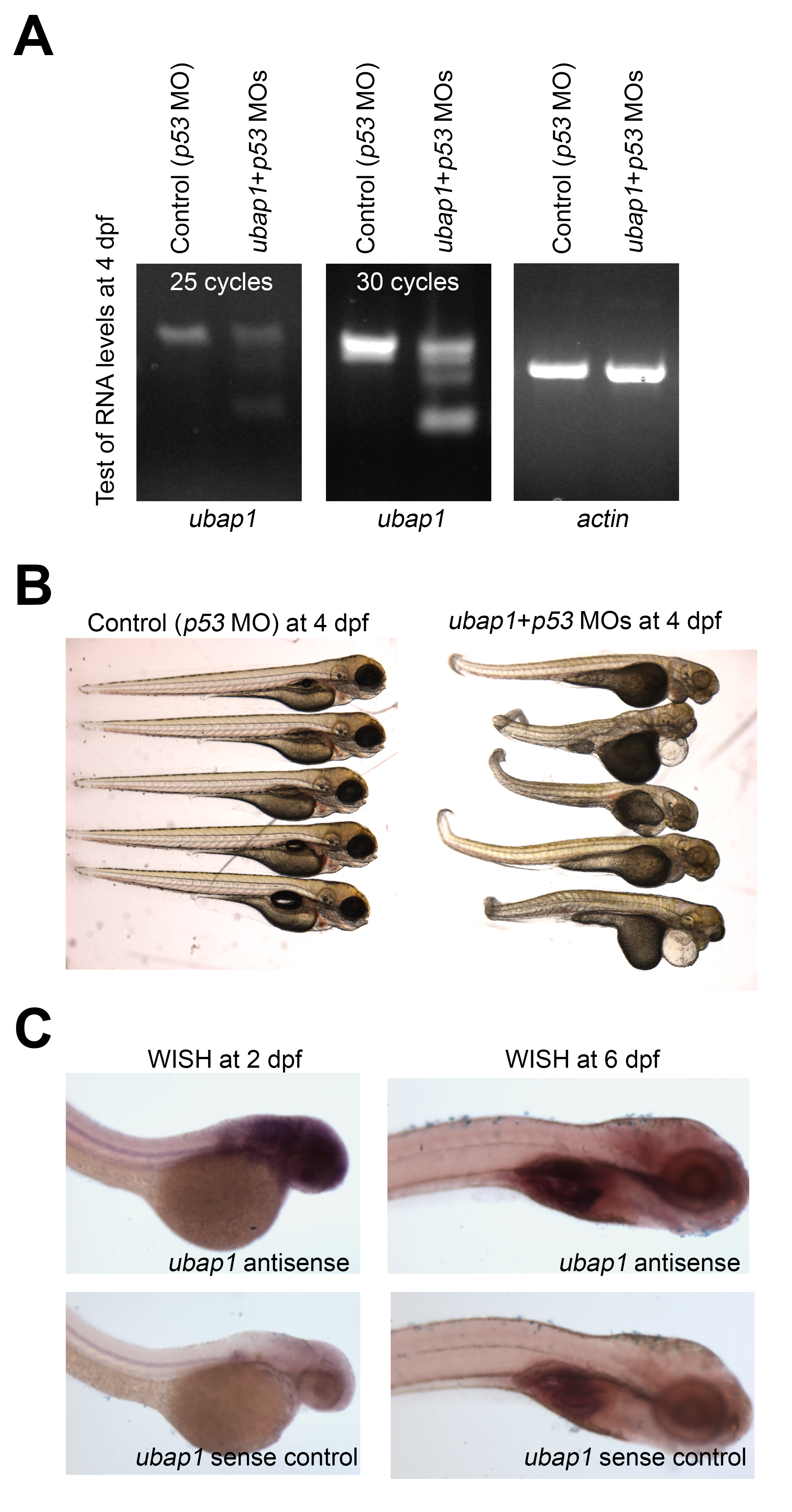

Supplement: S7 Fig — (A) RT-PCR analysis of ubap1 and actin expression in control and ubap1 morphants co-injected with p53 morpholino. (B) Brightfield images of groups of control and ubap1 morphants. Ubap1 morphants display widespread developmental defects, unlike fcc143 GFP-high embryos (see Fig 4B). (C) WISH of ubap1 antisense and sense probes in 2 and 6 dpf embryos. Ubap1 expression, shown by the antisense probe, was not detected in the caudal hematopoietic tissue or thymus in contrast to the GFP pattern in fcc143 carriers (see Fig 2). (TIF) [file pone.0131908.s007.tif]

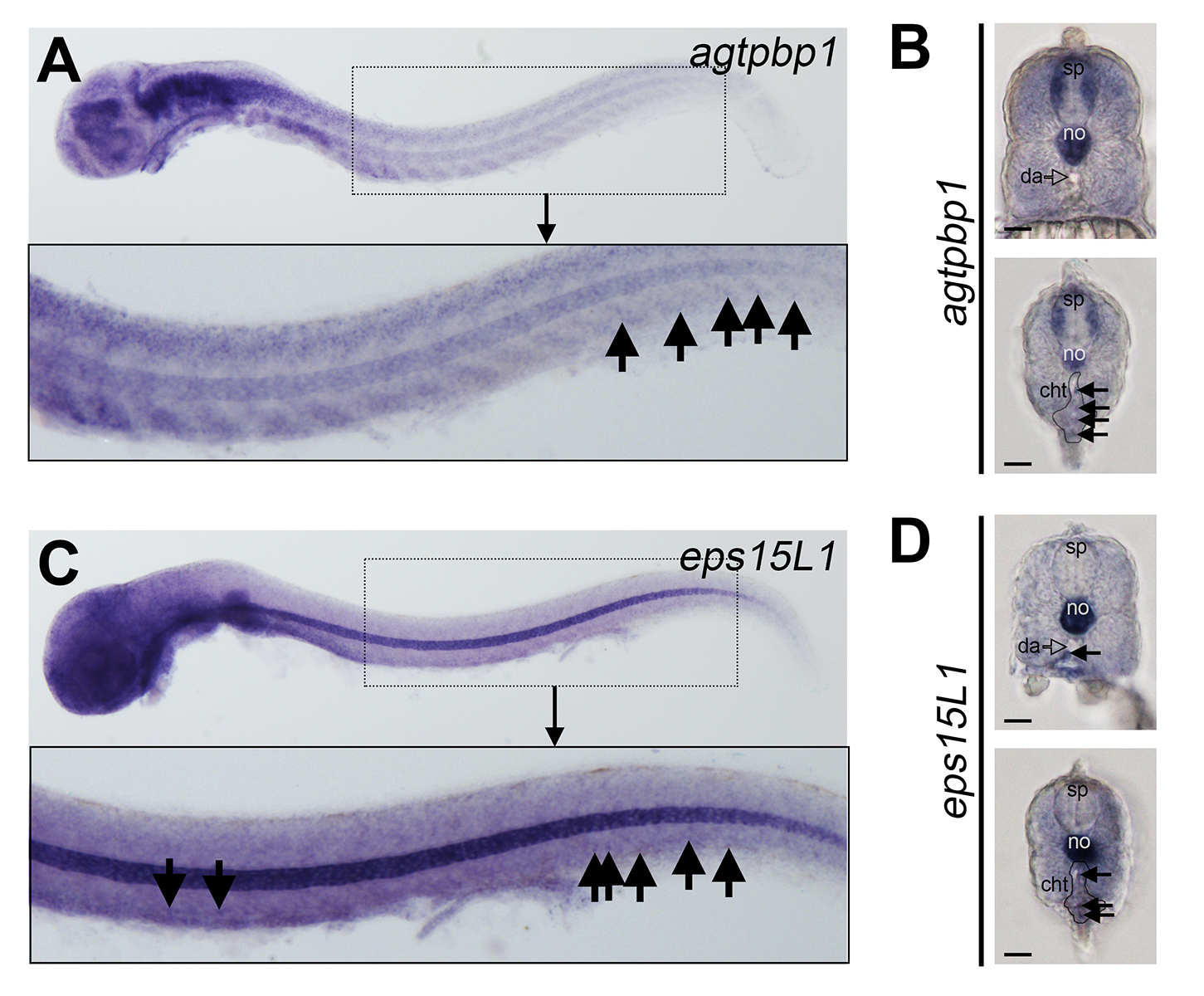

Supplement: S8 Fig — (A-D) WISH of the indicated genes in 2 dpf embryos. (A) Agtpbp1 expression is shown in purple. Image shows a lateral view of a representative embryo, anterior facing left. Embryo was deyolked. The boxed area is enlarged in the lower panel. Black arrows indicate agtpbp1-expressing cells in the caudal hematopoietic tissue (CHT). (B) Transverse sections through the trunk (top panel) and tail (bottom panel) regions to show agtpbp1 expression in a 2-dpf embryo. Black arrows in the lower panel indicate positive cells in the CHT. (C) Eps15L1 expression is shown in purple. Image shows a lateral view of a representative embryo, anterior facing left. Embryo was deyolked. The boxed area is enlarged in the lower panel. Black arrows indicate positive cells in the AGM and CHT. (D) Transverse sections through the trunk (top panel) and tail (bottom panel) regions to show eps15L1 WISH analysis in a 2-dpf embryo. Black arrows indicate positive cells in the ventral wall of the dorsal aorta (da; AGM region) and CHT in the top and bottom panels, respectively. sp = spinal cord, no = notochord, da = dorsal aorta, cht = caudal hematopoietic tissue region. (TIF) [file pone.0131908.s008.tif]

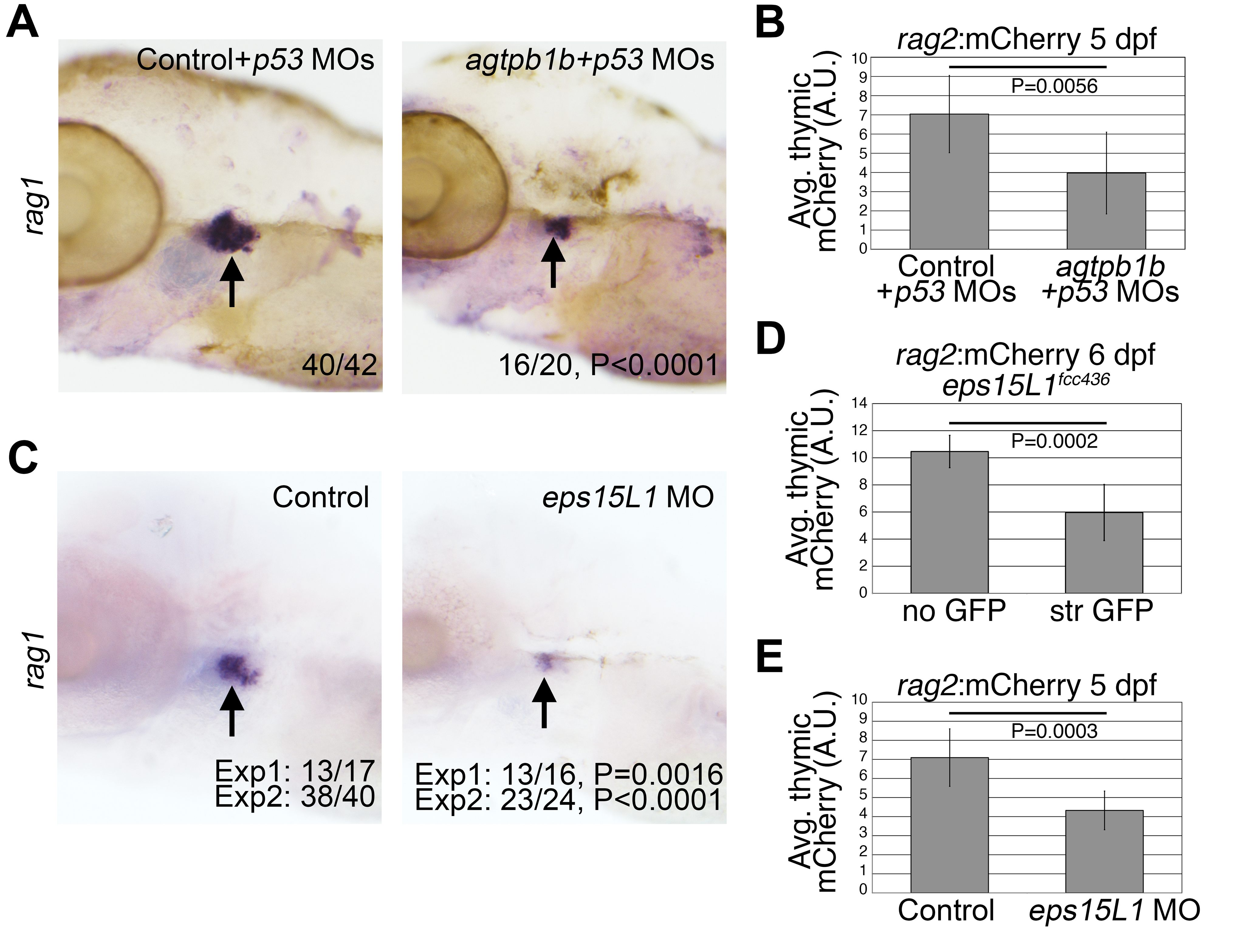

Supplement: S9 Fig — (A) WISH of rag1 in 5 dpf control and agtpbp1 morphants co-injected with p53 morpholino. N is indicated. Images show lateral views of the left side of the head. Arrows indicate rag1-expressing cells in the thymus. (B) Levels of rag2:mCherry transgene expression in the thymus of 5 dpf control and agtpbp1 morphants. Images of mCherry (B,D,E) in siblings were acquired using identical exposure settings. Fiji was used to quantify the whole mount expression from the acquired images (shown in S10 Fig). (C) WISH of rag1 in 5 dpf control and eps15L1 morphants. N is indicated. Two independent experiments were performed. Images show lateral views of the left side of the head. Arrows indicate rag1-expressing cells in the thymus. (D-E) Quantitation of the rag2:mCherry transgene expression in the thymus in lines eps15L1 fcc436-P1 (436) siblings at 6dpf (D) and control and eps15L1 morphants at 5 dpf (E). P values for mCherry quantitation were determined using two-tailed Student’s T-test; P values for WISH were determined using Fisher’s exact test. (TIF) [file pone.0131908.s009.tif]

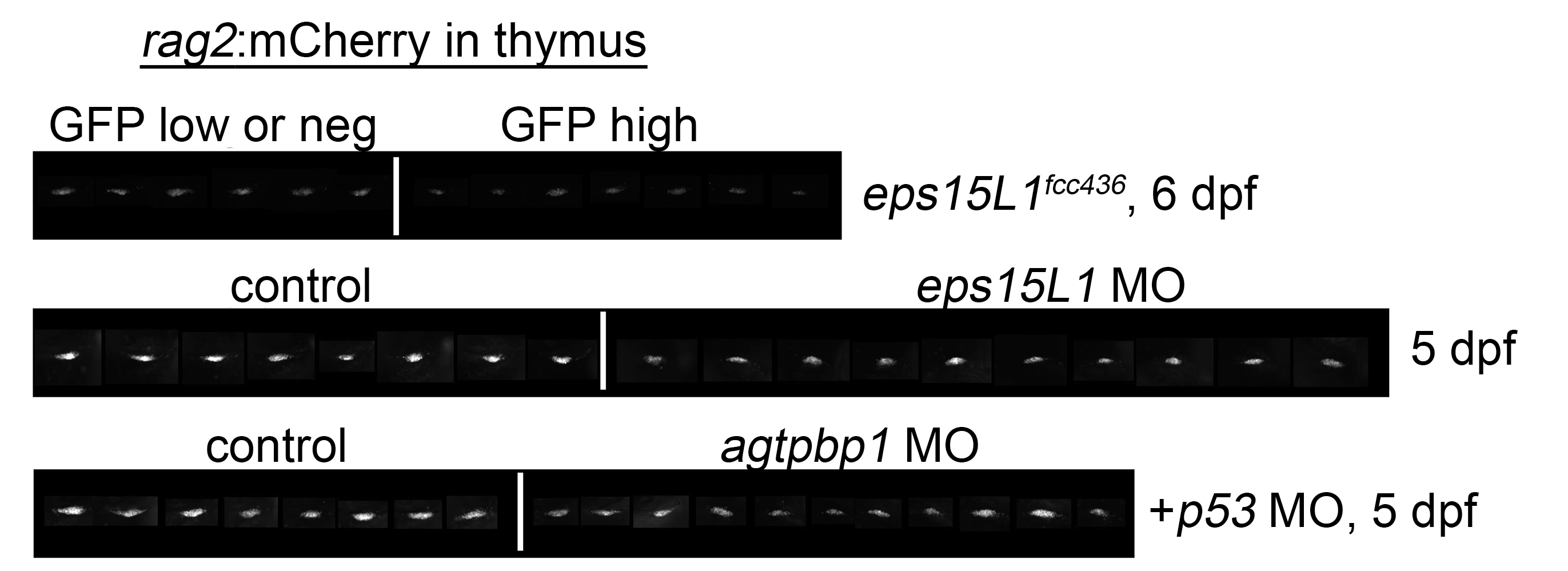

Supplement: S10 Fig — Images of the thymus in individual siblings in an experimental set are shown. Images of control and gene deficient embryos were acquired using identical exposure parameters. Each thymus image represents a different embryo. The embryos and stages are indicated. (TIF) [file pone.0131908.s010.tif]

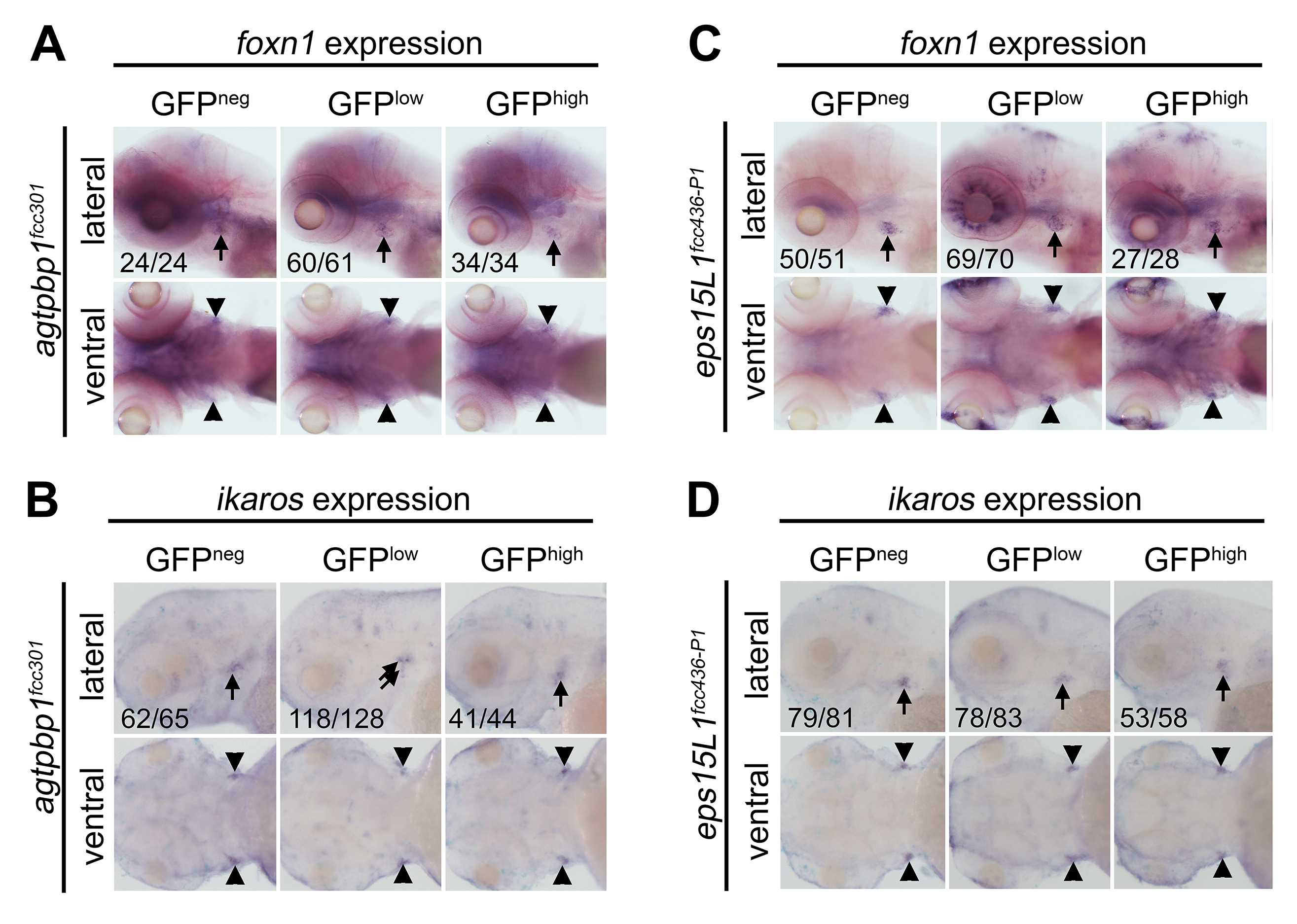

Supplement: S11 Fig — (A) WISH of foxn1 in 5 dpf agtpbp1 fcc301 siblings sorted prior to fixation by their level of GFP expression, although there was a range of GFP expression levels in this line. (B) WISH of ikaros in 3 dpf agtpbp1 fcc301 siblings separated prior to fixation based on their GFP expression level. (C) WISH of foxn1 in 5 dpf eps15L11 fcc436-P1 siblings displaying the indicated GFP expression level. (D) WISH of ikaros in 3 dpf eps15L11 fcc436-P1 siblings sorted prior to fixation by their level of GFP expression. Orientation, GFP expression levels and N are indicated. Neg = negative. Black arrows/arrowheads indicate WISH+ cells in the thymus. (TIF) [file pone.0131908.s011.tif]

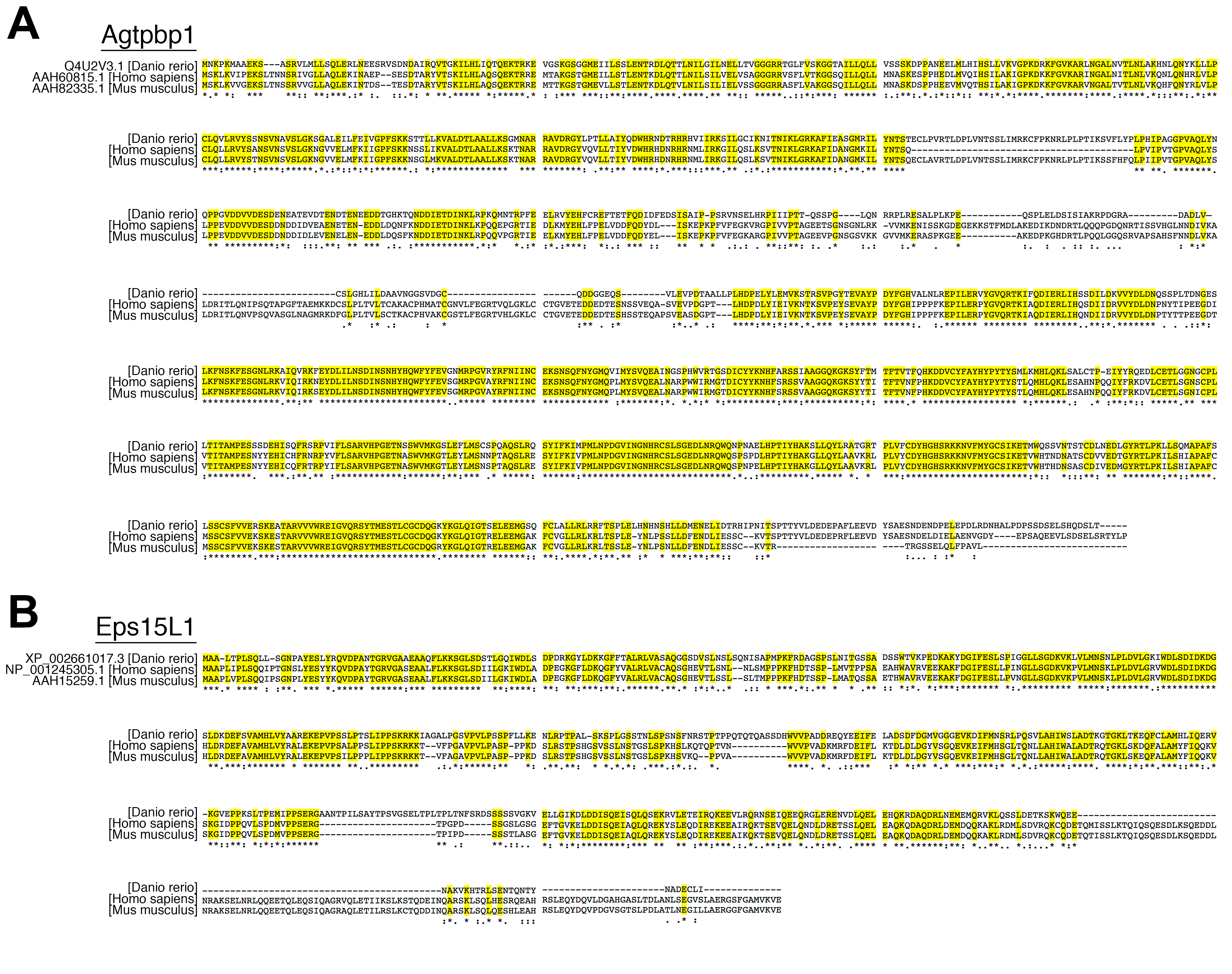

Supplement: S12 Fig — (A) Alignment of amino acid sequence of Agtpbp1 from Danio rerio, Homo sapiens and Mus musculus. (B) Alignment of amino acid sequence of Eps15L1 from Danio rerio, Homo sapiens and Mus musculus. (A-B) Clustal analysis; identical amino acids are highlighted; accession numbers of the proteins are listed, zebrafish proteins are predicted. Clustal Format alignment was generated through the www.phylogeny.fr site. (TIF) [file pone.0131908.s012.tif]

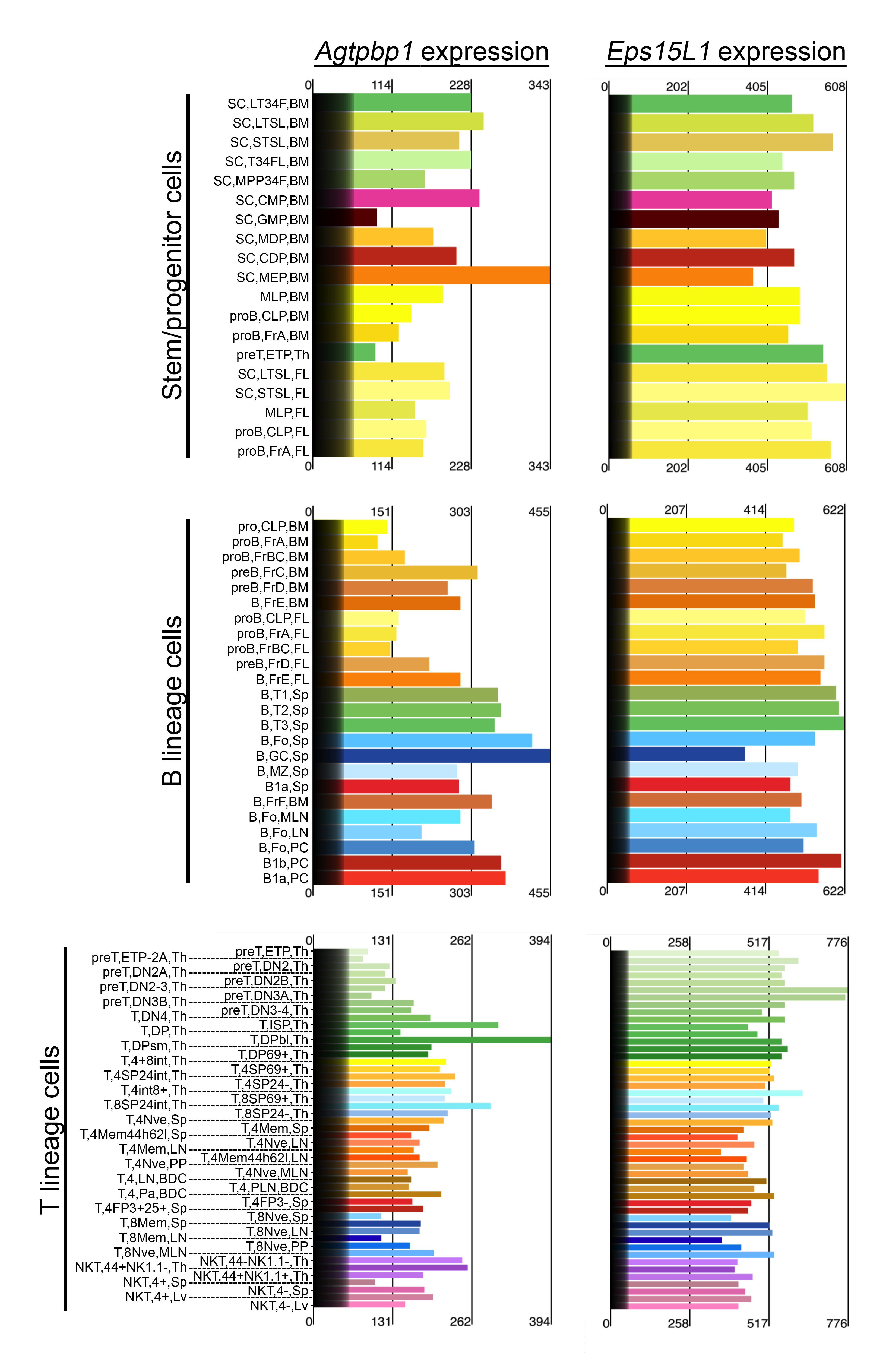

Supplement: S13 Fig — Gene skyline generated expression profiles of Agtpbp1 and Eps15L1 in purified hematopoietic populations as indicated. ImmGen = Immunological Genome Project, http://www.immgen.org/. (TIF) [file pone.0131908.s013.tif]
