# Supplementary material for: Mutagenesis Screen Identifies agtpbp1 and eps15L1 as Essential for T lymphocyte Development in Zebrafish
Source: PLoS One. 2015 Jul 10;10(7):e0131908. doi: 10.1371/journal.pone.0131908 (PMC4498767; doi:10.1371/journal.pone.0131908)
Supplement: S1 Table — The fcc line number, target gene, sequence of the oligomer and experimental use are indicated. (PDF) [file pone.0131908.s014.pdf]

Supplementary S1 Table. List of primer sequences.

**Line #**	**Gene**	**Primer**	**Use**
24	*ralgds*	5'-ATTTGGCCTCTTGGTGTTTC	genotyping (intron)
88 P1	*vps4b*	5'-TGGCTGCCAACAACAACTTA	genotyping (intron)
301	*agtpbp1*	5'-CCAAGAGAACGCACTGTAAGGAGTTCAG	genotyping (coding/cDNA)
403	*vps35*	5'-GCATTAGCCTGGCTTTTTCA	genotyping (intron)
420	dnajb14	5'-AGTTCCTGGCTTTACCACTGA	genotyping (intron)
436	*eps15L1*	5'-CAGGAAATATGGCGGCGCTCAC	genotyping (exon)
559	*adamts3*	5'-CAAGCTTAGCAGGGACAAGC	genotyping (intron)
629	*eif3c*	5'-AAGCGTTTTCTGTGTGCTGT	genotyping (intron)
667	*abi1a*	5'-CGTATTTGGTAATCACCGATTTT	genotyping (intron)
688	*hnrpkl*	5'-AAACGTGGAATGGCTTGTTC	genotyping (intron)
	gal4 bp152 (used with primers to *ralgds, agtpbp1, vps35, dnajb14, eps15L1, adamts3, eif3c, abi1a* and *hnrpkl*)	5'-GCCCTAGTCAGCGGAGACCTTTTGGT	RACE and genotyping (cDNA and DNA)
418	*ric8a*	5'-ACCAGCAACCTTCTTGCAGT	genotyping (intron)
626	*exosc*	5'-GCCACTTGTTGGTTTGGAGT	genotyping (intron)
	UAS (used with primers to *exosc* and *ric8a*)	5'-CTTCTAATCCGTTGCCGGAGGACTGT	genotyping (intron)
	glis1b forward (control for genomic DNA)	5'-CTCTGAGGTTGCCAGCTCTT	genotyping control (intron)
	glis1b reverse (control for genomic DNA)	5'-AGCAGGTTGTTGAAGCCAGT	genotyping control (intron)
	iP 3'end B4-left 1	5'-CTGTACAAGTAAAGCGGCCGCGACTC	iPCR
	iP 3'end B4-left 2 nested	5'-ATGTTTCAGGTTCAGGGGGAGGTGTG	iPCR
	iP 3'end B4-right 1	5'-CTTCTAATCCGTTGCCGGAGGACTGT	iPCR
	iP 3'end B4-right 2 nested	5'-TGAATTCCATGGATCCGGAGATCTGA	iPCR
	iP 5'end B4-left 1	5'-CATGTGACCATGTGGAGTCAGCTTCC	iPCR
	iP 5'end B4-left 2 nested	5'-CGCTAGTGGGTGGCATGCTGATAACTT	iPCR
	iP 5'end B4-right 1	5'-AGGTCTCCGCTGACTAGGGCACATCT	iPCR
	iP 5'end B4-right 2 nested	5'-GATGCCGTCACAGATAGATTGGCTTCAG	iPCR
	gal4 bp99	5'-GTTCTTCAGACACTTGGCGCACTTCG	RACE
	agtpbp1-exon 3 forward	5’-TCCCGAGTGCTGATGTTGCTGAGTCA	RT-PCR
	agtpbp1-exon 5 reverse	5’-TCAAAATCCCAAGGATGTTCAAGGTG	RT-PCR
	eps15L1-exon 15 forward	5’-TGCGACAGAGGAACAGCGAAATCCAG	RT-PCR
	eps15L1-exon 16 reverse	5’-GTTTCACCTTGGCGTTCTCCTCCTGC	RT-PCR
	igH-μ-forward	5'-CCTCCTCAGACTCTGTGGTGA	RT-PCR
	igH-μ-reverse	5'-TTGCTGATCCACCTTCTAATTC	RT-PCR
	lck-forward	5'-AGATGAATGGTGTGACCAGTGTA	RT-PCR
	lck-reverse	5'-GATCCTGTAGTGCTTGATGATGT	RT-PCR
	β-actin-forward	5'-GCCAACAGGGAAAAGATGACACAG	RT-PCR
	β-actin-reverse	5'-GAGTATTTACGCTCAGGTGGGGC	RT-PCR
	agtpbp1 forward	5'-CCAAGAGAACGCACTGTAAGGAGTTCAG	cloning
	agtpbp1 reverse	5'-TAATACGACTCACTATAGGG	cloning
	eps15L1 forward	5'-CAGGAAATATGGCGGCGCTCAC	cloning
	eps15L1 reverse	5'-TAATACGACTCACTATAGGG	cloning
	agtpbp1	5’-TTGTGTGTGGCAGTACCTCAAGAGA	Morpholino
	eps15L1	5’-ACACGGTGTCAGTGTCTCACCATAT	Morpholino
	ubap1	5'-AGGAAGGAAATCAGACTTACTTGCA	Morpholino
	control morpholino	5'-CCTCTTACCTCAGTTACAATTTATA	Morpholino, Standard (negative) control oligo from GeneTools (human *β-globin*)
