# Supplementary material for: Mutagenesis Screen Identifies agtpbp1 and eps15L1 as Essential for T lymphocyte Development in Zebrafish
Source: PLoS One. 2015 Jul 10;10(7):e0131908. doi: 10.1371/journal.pone.0131908 (PMC4498767; doi:10.1371/journal.pone.0131908)
Supplement: S2 Table — (PDF) [file pone.0131908.s015.pdf]

Supplementary S2 Table. Summary of embryonic GFP expression in lines analyzed by FACS and results from RT-PCR analysis of GFP^{+} cells from 6 week old juvenile fish

Expression pattern group	Line*	GFP expression pattern	Insertion in gene:	Entrez ID	Method	Chr.	Position	Strand	Exon rank	RT-PCR signal
**Hematopoietic**	*Tg(GBT-B4)fcc24*	2dpf: PBI, circulation	*ralgds*	799483	lmPCR	8	31822987	-	2	low cell #
	vps4b$^{fcc88-P1Gt}$	2/6dpf: skin, circulation	*vps4b*	393880	lmPCR	2	13060212	-	2	*lck*
	Tg(GBT-B4)fcc143	2dpf: AGM, PBI; 6dpf: thymus epithelium	*(ubap1)*	28279726	RACE	21	n.a.	n.a.	6	low cell #
	agtpbp1^{fcc301Gt}	2/6dpf:AGM, kidney, nerves, circulation	*agtpbp1*	65335310	RACE	8	n.a.	n.a.	2	negative
	Tg(GBT-B4)fcc337-P2	6dpf: thymus								*igH-μ, lck*
	vps35^{fcc403Gt}	2dpf: PBI, blood vessels, notochord; skin; 6dpf:circulation,intestine, liver	*vps35*	561697	lmPCR	7	44541300	-	2	*igH-μ, lck*
	eps15L1$^{fcc436-P1Gt}$	2dpf: vessels, PBI, AGM, hatching gland, skin; 6dpf: pancreas,distal intestine, kidney, skin, vessels, circulation	*eps15L1*	528472310	RACE	2	n.a.	n.a.	2	*igH-μ, lck*
	Tg(GBT-B4)fcc510	2dpf: PBI, notochord, kidney; 6dpf: notochord, kidney, enteric nervous system								negative
	Tg(GBT-B4)fcc522	2dpf: skin, PBI; 6dpf: vessels, intestine, circulation								*igH-μ, lck*
	adamts3^{fcc559Gt}	2dpf: cells in AGM; 6dpf: cells in anterior kidney	*adamts3*	568788	lmPCR	5	47079485	+	15	low cell #
	abi1a^{fcc667Gt}	2dpf: blood vessels, PBI; 6dpf: vessels, circulation	*abi1a*	393711	lmPCR	24	6019688	+	2	*lck*
	Tg(GBT-B4)fcc688	2dpf: PBI, skin, kidney, nose, notochord; 6dpf: skin, kidney, spinal chord, hair cells (ear), intestine	*hnrpkl*	406264	lmPCR	5	56989741	-	11	n.d.
**Includes kidney**	*Tg(GBT-B4)fcc313*	2dpf: kidney tubules; 6dpf: liver ducts								low cell #
	Tg(GBT-B4)fcc360-P3	2/6dpf: anterior kidney tubules								low cell #
	Tg(GBT-B4)fcc363-P2	6dpf: kidney (corpuscles of Stannius)								low cell #
	Tg(GBT-B4)fcc27	2/6dpf: kidney, blood vessels in head	candiate: *slc2a1*	555778	lmPCR	23	22496878	+	2	n.d.
	Tg(GBT-B4)fcc474P1	2dpf:skin, eye; 6dpf: neural tube, eye, kidney								*igH-μ, lck*
**Ubiquitous**	*Tg(GBT-B4)fcc118*	2/6dpf: ubiquitous								*lck*
	Tg(GBT-B4)fcc88-P2	2/6dpf: weak ubiquitous								low cell #
	Tg(GBT-B4)fcc337-P1	2/6dpf: strong ubiquitous								*igH-μ, lck*
	Tg(GBT-B4)fcc618	2dpf: weak ubiquitous; 6dpf: no GFP								n.d.
	Tg(GBT-B4)fcc711	2/6dpf: ubiquitous								*igH-μ, lck*
**Other patterns**	*Tg(GBT-B4)fcc57-70*	6dpf: skin cells, anus, ear								n.d.
	Tg(GBT-B4)fcc144-P1	6dpf:posterior eye, brain, dorsal muscles, gills	candidate: *tead3a*	386763	lmPCR	23	5666933	-	5	negative
	Tg(GBT-B4)fcc144-P2	6dpf: muscle, liver, intestine, skin								*igH-μ*
	Tg(GBT-B4)fcc144-P3	6dpf: heart and trunk muscle								low cell #
	Tg(GBT-B4)fcc316	2dpf: nasal epithelium; 6dpf: weak								low cell #
	Tg(GBT-B4)fcc322	6dpf: middle of intestine								n.d.
	Tg(GBT-B4)fcc360-P1	6dpf: cells of ear canals								low cell #
	Tg(GBT-B4)fcc360-P2	6dpf: notochord, canal under distal ear								low cell #
	Tg(GBT-B4)fcc363-P1	2/6dpf: intestine	*exosc3*	565000	lmPCR	14	53499045	+	1	*igH-μ, lck*
	Tg(GBT-B4)fcc374-P2	6dpf: ear canal, nose, notochord								low cell #
	Tg(GBT-B4)fcc374-P1	6dpf: forebrain, tectum								low cell #
	Tg(GBT-B4)fcc382-P1	2dpf: neural tube, blood vessels or nerves (?); 6dpf: nerves in hindbrain								*igH-μ, lck*
	Tg(GBT-B4)fcc382-P2	6 dpf: nerves in brain, vagus nerve, jaw								n.d.
	Tg(GBT-B4)fcc413	6dpf: bones/cartiladge in jaw								n.d.
	Tg(GBT-B4)fcc418-P1	2dpf: epiphysis, dorsal eye, hindbrain, spinal cord; 6dpf: brain, hypophysis, eye	*ric8a*	449546	lmPCR	25	10677814	+	3	*igH-μ, lck*
	Tg(GBT-B4)fcc418-P2	2dpf: stripe in brain								low cell #
	Tg(GBT-B4)fcc420	6dpf: trunk muscle, cells around heart	*dnajb14*	792752	lmPCR	1	26842202	+	2	*igH-μ, lck*
	Tg(GBT-B4)fcc423-P1	2dpf: retina fusion zone; 6dpf: subset of photo receptor cells								*igH-μ*
	Tg(GBT-B4)fcc423-P2	2 dpf: nerves between eyes								low cell #
	Tg(GBT-B4)fcc436-P2	6dpf: muscle jaw trunk								low cell #
	Tg(GBT-B4)fcc436-P3	2/6dpf: skin cells								low cell #
	Tg(GBT-B4)fcc443	2dpf: forebrain; 6dpf: no GFP expression	candidate: *clybl*	641475	lmPCR	9	32246199	+	2	low cell #
	Tg(GBT-B4)fcc458	2dpf: notochord; 6dpf: no GFP expression								low cell #
	Tg(GBT-B4)fcc460	2/6dpf: skin cells								negative
	Tg(GBT-B4)fcc475	2dpf: forebrain; 6dpf: cells in brain								low cell #
	Tg(GBT-B4)fcc516	2dpf: brain (weak); 6dpf: neurons in eye								*lck*
	Tg(GBT-B4)fcc629	2dpf: trunk muscle, brain; 6dpf: ubiqitous, prominent thymus expression	*eif3c*	334234	lmPCR	12	4637151	-	8	*lck*
	Tg(GBT-B4)fcc634	2/6dpf: skin on head								n.d.
	Tg(GBT-B4)fcc652	2dpf: neural tube, motoneuron; 6dpf: motoneuron, enteric nervous system, hair cells								low cell #
	Tg(GBT-B4)fcc683	2dpf: forebrain, eye								negative

*GBT-B4 = Gt(LOXP-GAL4-VP16-FRT, syUAS:EGFP-FRT-LOXP)

Genes confirmed by linkage, but not determined to change expression of target are listed using Tg(GBT-B4)fcc nomenclature

n.d. = not determined, negative for GFP+ cells by FACS; low cell# = few GFP+ cells, could not detect robust *β-actin* band by RT-PCR

Candidate = lines where linkage to GFP+ embryos was not confirmed
