# Supplementary material for: Mutagenesis Screen Identifies agtpbp1 and eps15L1 as Essential for T lymphocyte Development in Zebrafish
Source: PLoS One. 2015 Jul 10;10(7):e0131908. doi: 10.1371/journal.pone.0131908 (PMC4498767; doi:10.1371/journal.pone.0131908)
Supplement: S3 Table — (PDF) [file pone.0131908.s016.pdf]

Supplementary S3 Table. Summary of WISH analysis in mutants from the indicated lines

**Line***	**42 hpf *cmyb***	**48 hpf *mpx***	**48 hpf *hbae1***	**d7 *mpx***	**d7 *hbae1***	**d4-7 *lck***
Tg(GBT-B4)fcc24	normal	normal	normal	normal	normal	normal
vps4b$^{fcc88\text{-}P1Gt}$	normal	normal	normal	n.d., lethal between day 3 and 4	n.d.	n.d.
Tg(GBT-B4)fcc143	normal	normal	normal	normal	normal	decreased on d4, 5 and 6
agtpbp1^{fcc301Gt}	normal	normal	normal	normal	normal	decreased or absent
Tg(GBT-B4)fcc337-P2	n.d.	n.d.	n.d.	normal	normal	decreased or absent on d4
vps35^{fcc403Gt}	normal	normal	normal	n.d., edema on day 6	n.d.	n.d.
eps15L1$^{fcc436\text{-}P1Gt}$	normal	normal	normal	normal	normal	decreased
Tg(GBT-B4)fcc510	normal	normal	n.d.	normal	n.d.	normal
Tg(GBT-B4)fcc522	n.d.	normal	normal	normal	normal	normal
abi1a^{fcc667Gt}	normal	normal	n.d.	normal	n.d.	normal
Tg(GBT-B4)fcc688	normal	normal	normal	n.d.	n.d.	n.d.

**GBT-B4 = Gt(LOXP-GAL4-VP16-FRT, syUAS:EGFP-FRT-LOXP)*
